# Dynamic role of gastric stem cells and chief cells in precancerous lesions of gastric cancer: global knowledge mapping and emerging trends based on bibliometric analysis from 2004 to 2024

**DOI:** 10.3389/fonc.2025.1556009

**Published:** 2025-05-16

**Authors:** Chen Wang, Lijie Zhou, Yangang Wang

**Affiliations:** ^1^ The Third Clinical Medical College of Beijing University of Chinese Medicine, Beijing, China; ^2^ The Department of Spleen and Stomach, The Third Affiliated Hospital of Beijing University of Chinese Medicine, Beijing, China; ^3^ Department of Spleen and Stomach II, Hebei Province Hospital of Traditional Chinese Medicine, Hebei, Shijiazhuang, China

**Keywords:** gastric stem cell, chief cell, precancerous lesions of gastric cancer, bibliometric analysis, visualization

## Abstract

**Background:**

Gastric stem cells (GSCs) and chief cells are vital for maintaining gastric epithelial homeostasis. However, under pathological conditions, these cells undergo significant functional changes, contributing to the progression of precancerous lesions of gastric cancer (PLGC). Dysregulation of key signaling pathways such as WNT, NF-κB, and YAP leads to aberrant cellular behaviors, which are implicated in the early stages of gastric carcinogenesis. This study aimed to elucidate the roles of GSCs and chief cells in maintaining gastric epithelial integrity, their contributions to the development of precancerous lesions, and the molecular mechanisms that regulate their behavior during disease progression.

**Methods:**

The study integrated bibliometric analysis, pathfinding, and data visualization using tools such as CiteSpace, VOSviewer, and R software. Functional enrichment of target genes was analyzed using KEGG and GO databases. The study focused on gastric cell changes, including differentiation, dedifferentiation, and signaling pathway activation, within the context of GSC and chief cell plasticity. Molecular markers and pathway-specific mechanisms were analyzed to clarify their contributions to gastric precancerous lesions.

**Results:**

Data from the WoSCC database from 2004 to 2024 showed a steady increase in publications on “PLGC-gastric stem cells” and “PLGC-chief cells,” with the United States, China, and Japan leading in publication volume. International cooperation was evident, particularly with Canada playing a central role in academic exchanges. Key terms included stem cells, intestinal chemotaxis, and cancer, with recent focus on spasmolytic polypeptide-expressing metaplasia.

**Conclusion:**

The dynamic interactions between GSCs and chief cells are fundamental to gastric homeostasis and disease progression. GSCs primarily drive chronic inflammation-induced metaplasia and dysplastic changes, while chief cells facilitate acute epithelial repair through dedifferentiation. These findings highlight potential therapeutic targets and emphasize the importance of regulating dysregulated pathways to prevent gastric cancer. The research results will guide future studies in the fields of “PLGC-gastric stem cells” and “PLGC-chief cells,” focusing on the spatiotemporal dynamics of each cell type under various injury and inflammatory conditions, as well as identifying early biomarkers of cellular changes for timely intervention.

## 1 Introduction

Gastric cancer (GC) is a globally prevalent malignant tumor of the gastrointestinal tract, with more than 968,000 new cases and nearly 660,000 deaths already in 2022, ranking fifth in the world in terms of both cancer incidence and mortality (1). The Correa evolutionary pattern of gastric adenocarcinogenesis is widely recognized by the academic community: under external triggers such as Helicobacter pylori (H. pylori) infection, the gastric mucosa undergoes a series of pathological transitions from a normal state to chronic gastritis (CG), chronic atrophic gastritis (CAG), intestinal metaplasia (IM), dysplasia (Dys)/intraepithelial neoplasia (IN), and ultimately GC (2). This sequence forms the core pathway of the “inflammation-to-cancer” transformation. Precancerous lesions of gastric cancer (PLGC) represent an intermediate stage between chronic inflammation and malignancy, encompassing CAG, IM, and dysplasia. These lesions exhibit bidirectional progression, wherein appropriate medical intervention can either arrest or reverse the process, preventing malignant transformation (3). Given the rising global burden of gastric cancer, early detection and therapeutic targeting of PLGC have become critical components of secondary prevention (4). Gastric mucosal damage can be categorized into focal and diffuse types. Focal damage, often caused by toxin exposure, bile reflux, or specific infections, is characterized by an unchanged cellular differentiation pattern and repairability, presenting as focal erosions or full-thickness ulcers. These lesions rely on the proliferation of adjacent gastric mucosal cells and surface cell migration for rapid repair. In contrast, diffuse damage involves abnormal differentiation of gastric mucosal cells and is a chronic process, including spasmolytic polypeptide-expressing metaplasia (SPEM) and IM. These changes are induced by factors such as Trefoil factor 2 (TFF2) or Cdx2 and are considered early markers of GC (4).

The cancer stem cell (CSC) theory offers a novel perspective for understanding the origin, metastasis, high recurrence rates, and chemotherapy resistance of cancer (5). Tumors comprise heterogeneous cell populations, but only a subset of CSCs possess differentiation potential, with their self-renewal ability driving tumor formation and malignant progression (6). In GC, the overexpression of specific stemness genes may enhance CSC self-renewal capacity and correlate closely with patient prognosis. Gastric stem cells (GSCs), usually located in the base or isthmus of gastric glands, are responsible for renewing and maintaining the gastric epithelium under physiological conditions (7). These stem cells differentiate into various gastric cell types, including mucous neck cells, parietal cells, and chief cells, to maintain the structural integrity of the gastric epithelium. However, under chronic inflammation or H. pylori infection, these stem cells may undergo abnormal differentiation, producing cells with intestinal epithelial characteristics (e.g., goblet cells), thereby promoting IM (8). SPEM, a specific type of metaplasia, expresses TFF2 and MUC6 in fundic glands and primarily originates from the transdifferentiation of mature chief cells, mucous cells, and isthmus stem cells. This reflects the reprogramming process of gastric mucosal epithelial cells, which can further progress to IM and eventually GC, making it a new focus for GC prevention and control (9). Gastric stem cells and chief cells play critical roles in the progression of PLGC. In-depth exploration of the origins and mechanisms of these two cell types is crucial for understanding the pathogenesis of PLGC and advancing secondary prevention of GC.

Bibliometrics, a tool that applies mathematical and statistical methods to analyze scientific literature, examines collaboration networks among authors, institutions, countries, journals, and keywords, providing qualitative and quantitative predictions of hotspots and trends in specific research fields (10). This analysis visually presents the structure and patterns of research literature through scientific mapping (11). To date, bibliometric analysis in the fields of “PLGC-gastric stem cell” and “PLGC-chief cell” remains unexplored. Therefore, this study aims to employ bibliometric software like CiteSpace (12) and VOSviewer (13) and R packages to comprehensively quantify the overall research landscape on “PLGC-gastric stem cell” and “PLGC-chief cell” over the past 20 years, providing directional recommendations for future studies.

## 2 Methods

### 2.1 Data source and study selection

**Figure 1:**
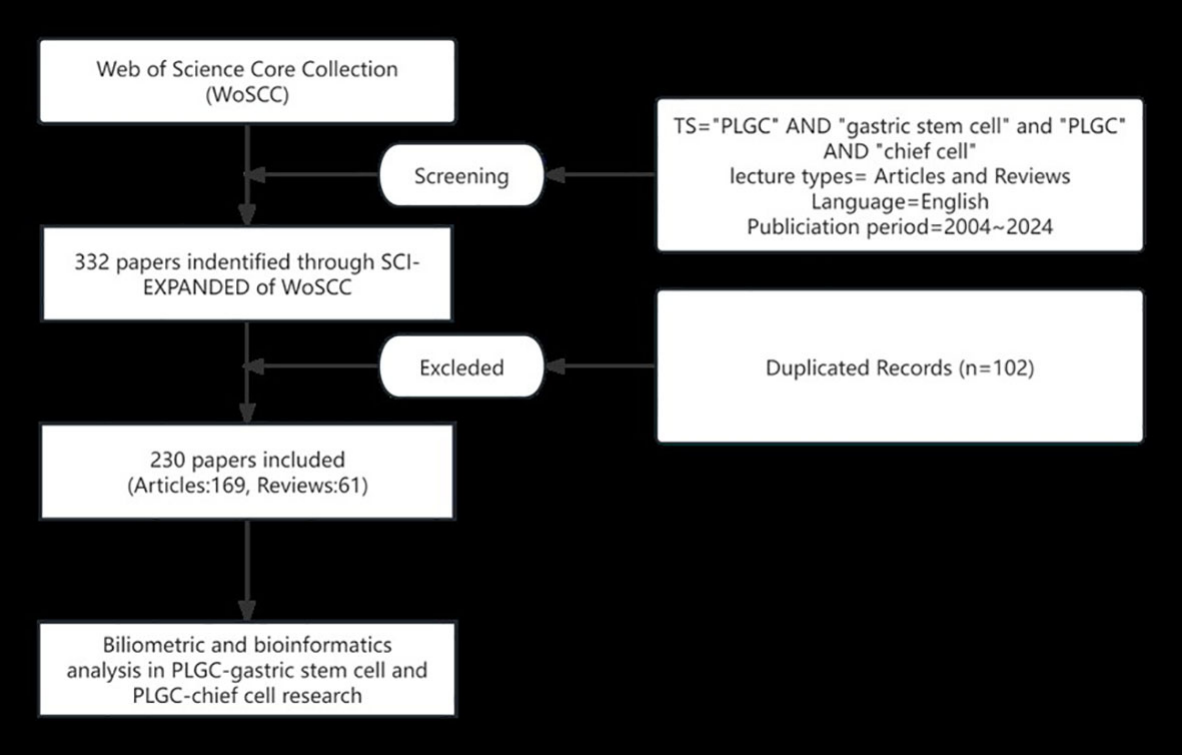
Literature search and screening flowchart.

Based on the precedence set by bibliometric research, the Web of Science Core Collection (WoSCC) was chosen as the primary database due to its extensive coverage of over ten thousand high-impact journals. Specifically, the Science Citation Index-Expanded within WoSCC was utilized for this investigation. To ensure comprehensiveness, two independent researchers conducted a thorough literature search for original research articles and reviews. The search strategy employed the following key terms: Search Term 1 (ST1): Topic (TS) = (“precancerous lesions of gastric cancer” OR “chronic atrophic gastritis” OR “intestinal metaplasia” OR “dysplasia” OR “intraepithelial neoplasia”); Search Term 2 (ST2): TS = (“gastric stem cell”); Search Term 3 (ST3): TS = (“chief cell”). The final search query combined these terms as follows: (ST1 AND ST2) OR (ST1 AND ST3), limiting the results to English-language publications published between January 1, 2004, and October 1, 2024. This meticulous approach yielded a total of 169 original articles and 61 reviews (**Figure 1**). To ensure comprehensive literature coverage, our search strategy explicitly included multiple synonyms for PLGC, such as “gastric intraepithelial neoplasia”, “intestinal metaplasia”, and “dysplasia”. A validation step was conducted, wherein independent researchers cross-checked the retrieved dataset against major systematic reviews in the field to confirm that no critical studies were omitted.

### 2.2 Data collection and refinement

This study meticulously extracted a core dataset from the WoSCC database to establish a solid foundation for subsequent in-depth analysis. The dataset included key information such as publication year, citation count, country or region of origin, research institution name, author details, source journal specifics, reference lists, and subject keywords. Next, duplicates in the data—such as country names, institution names, author names, and keywords—were carefully merged, spelling errors were corrected, and irrelevant terms were removed. The final, rigorously cleaned dataset was imported into Microsoft Excel 365 and specialized bibliometric visualization software for comprehensive bibliometric analysis (10).

### 2.3 In-depth analysis and visualization

To better understand these complex datasets, advanced techniques such as data visualization and clustering analysis were employed. For performance evaluation, metrics such as total publication count, total citation count, and average citation count per publication were integrated to comprehensively assess the publication and citation performance of authors, institutions, countries, and journals. Scientific network analysis focused on three key dimensions: (1) Author collaboration analysis revealed individuals or teams driving collective scholarly output, constructing detailed collaboration networks. (2) Co-citation analysis (the simultaneous citation of two articles by a third) provided insights into the evolution of core issues within the research field. (3) Keyword co-occurrence analysis examined the connections between research themes and identified potential future developments.

For visualizing scientific networks, CiteSpace 6.4.R1 (advanced version) and VOSviewer 1.6.20, both Java-based tools, were employed. These tools presented scientific outputs in the form of nodes, where nodes represent entities such as authors, institutions, countries, or keywords. The size of a node directly correlates with its publication count, reflecting the activity level of the entity, while edges between nodes represent relationships. Using CiteSpace 6.4.R1 and VOSviewer 1.6.20, we successfully visualized keyword co-occurrence, clustering, timelines, and time zones, as well as collaboration networks among countries, authors, and institutions, along with co-citation networks for references, journals, and authors.

### 2.4 Gene function analysis and pathway exploration

To uncover core signaling pathways and critical targets in the regulatory mechanisms of PLGC transformation, this study integrated advanced methods from the Gene Ontology (GO) classification system and the Kyoto Encyclopedia of Genes and Genomes (KEGG) pathway analysis (http://www.omicsbean.cn/). Comprehensive functional classification of the studied genes was performed, with biological processes involved scientifically predicted and rationally interpreted. To visually present the analysis results, carefully constructed visual charts were created. All reported enrichment analysis results underwent rigorous statistical testing, with adjusted p-values consistently below 0.05, ensuring the accuracy and reliability of the research conclusions.

## 3 Results

### 3.1 Overview of publication trends

**Figure 2:**
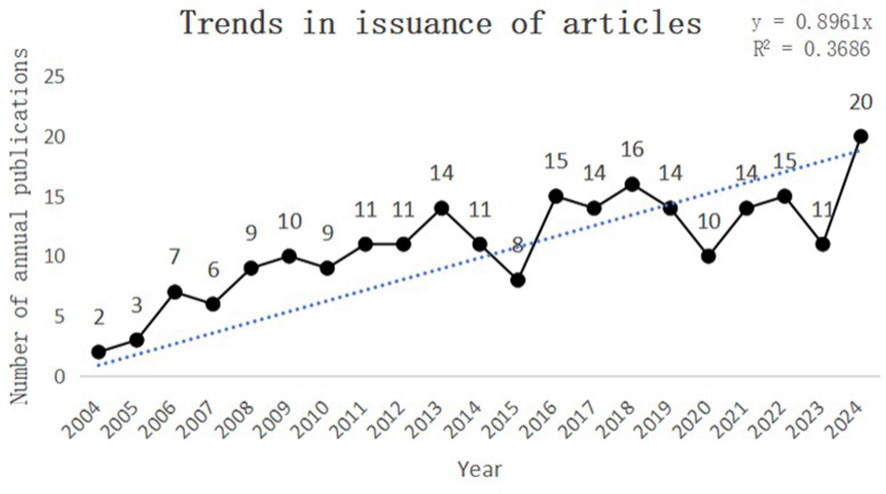
Trends in issuance of articles.

This study collected a total of 230 papers on the topics of “PLGC-gastric stem cell” and “PLGC-chief cell,” authored by 151 researchers from 156 research institutions across 31 countries and published in 131 different journals. As shown in **Figure 2**, the number of publications in this field has shown a steady upward trend from 2002 to 2024. Specifically, only two papers were published in 2004, whereas the number increased tenfold to 20 papers by 2024. This remarkable growth highlights the increasing popularity of this research field, underscoring its academic importance and societal impact.

### 3.2 Distribution of countries/regions and institutions

**Figure 3:**
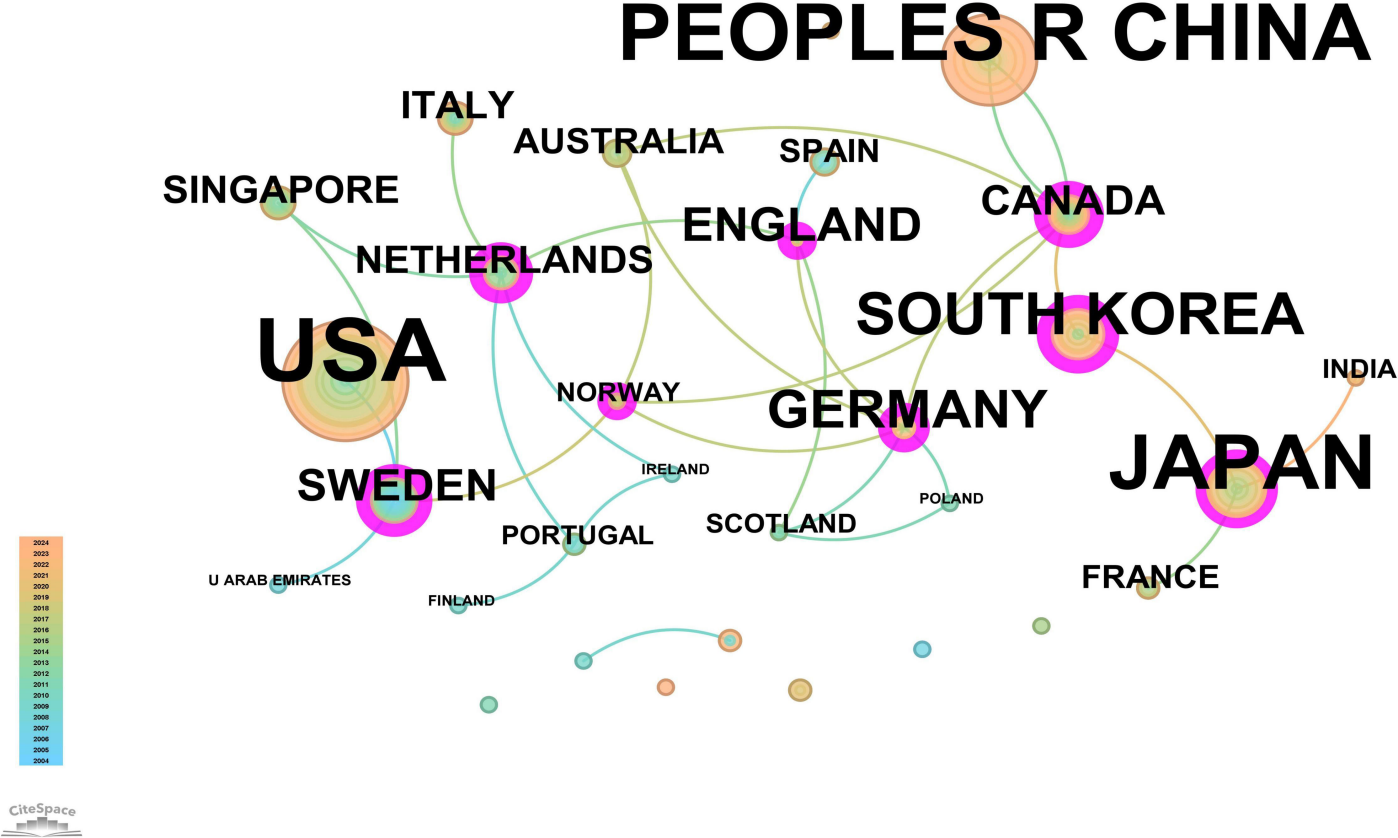
Network map of countries/regions for “PLGC-gastric stem cell” and “PLGC-chief cell” studies.

**Figure 4:**
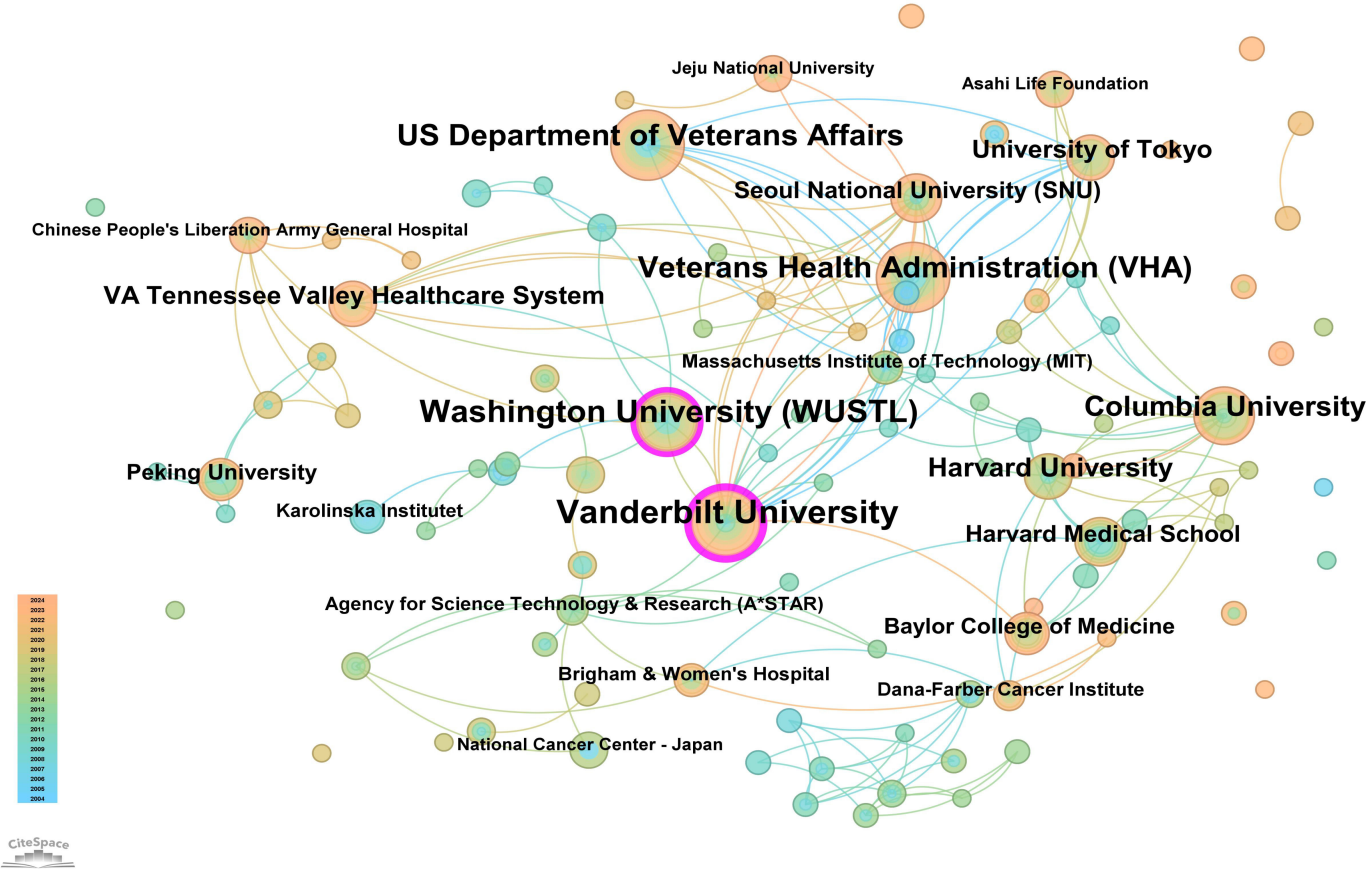
Institutional network map for “PLGC-gastric stem cell” and “PLGC-chief cell” studies.

The core journal papers in this study originated from 31 countries/regions and 156 institutions. **Supplementary Table 1** lists the top 10 countries/regions and institutions by publication count. The United States ranked first with 98 publications, followed by China with 62 publications, and Japan with 50 publications. At the institutional level, Vanderbilt University led with 21 publications, followed by Washington University (WUSTL) with 18, and the US Department of Veterans Affairs with 16 publications. A network mapping analysis was performed to reveal academic exchanges between countries/regions (**Figure 3**). The centrality of network nodes reflects the closeness of inter-country/regional collaboration. Generally, centrality greater than 0.1 indicates denser connections and higher correlation. The analysis showed that Canada had the highest centrality (0.39), serving as a critical bridge in international collaborations and academic exchanges. Other notable nodes with high collaboration levels included Germany (0.38), the Netherlands (0.36), England (0.34), South Korea (0.25), and Sweden (0.23). In terms of institutional collaboration, two closely cooperating clusters were identified, represented by Vanderbilt University and WUSTL, both with centrality values exceeding 0.1 (**Figure 4**). Notably, 8 out of the top 10 institutions by publication count are based in the United States, reflecting the country’s prominent role and significant contributions to research on gastric stem cells, chief cells, and precancerous gastric lesions.

### 3.3 Analysis of authors, citation frequency, and collaboration networks

**Figure 5:**
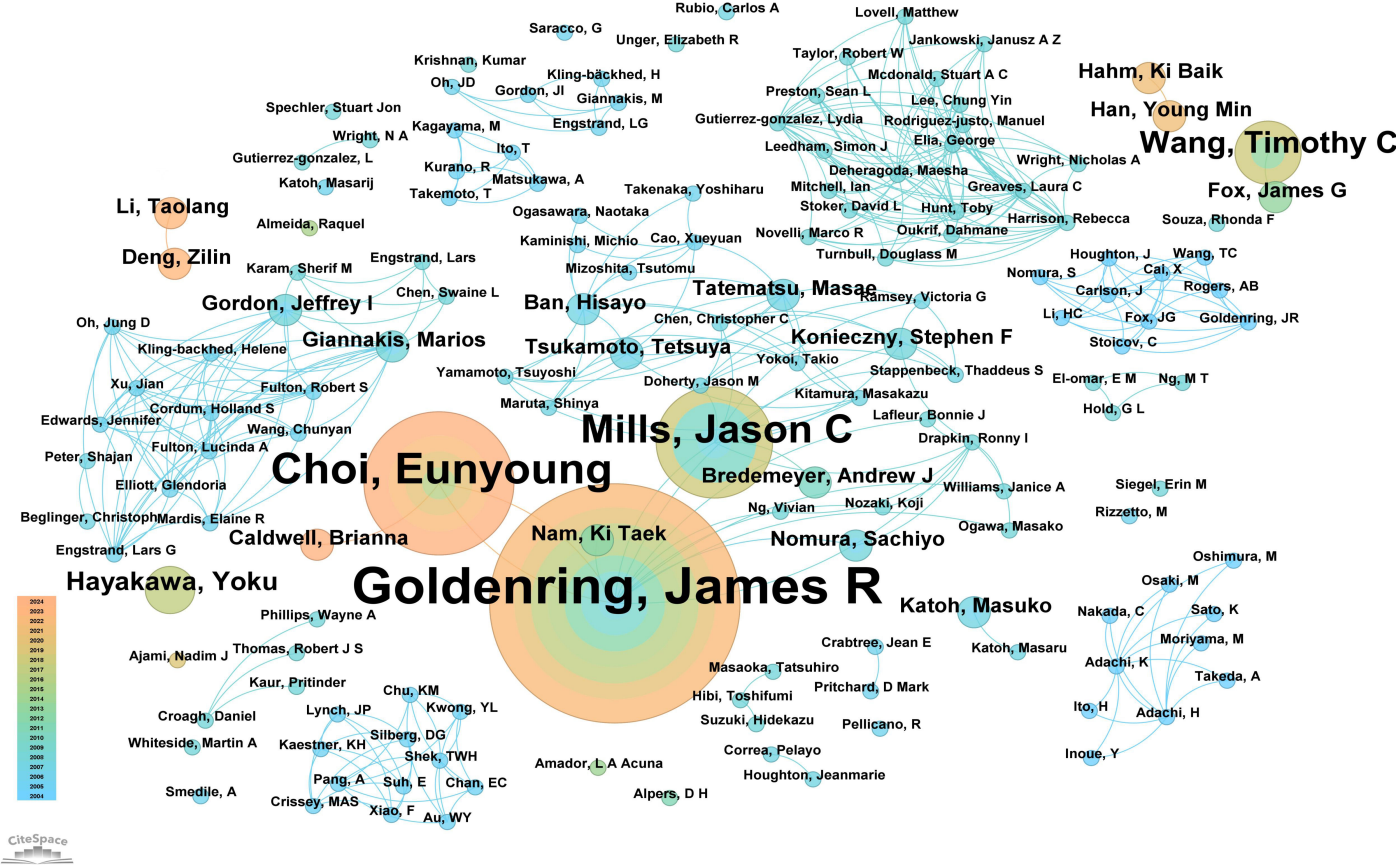
Network diagram of author collaborations for “PLGC-gastric stem cell” and “PLGC-chief cell” studies.

A total of 151 authors have contributed to research on “PLGC-gastric stem cell” and “PLGC-chief cell.” Of these, 130 authors (86.09%) published only one paper, demonstrating the broad participation of authors in this field. Sixteen authors (10.59%) published two papers, while only five authors (3.31%) published three or more papers, reflecting the depth and continuity of research in this field. **Supplementary Table 2** details the top 10 authors by publication count in this field. At the top were Professors James R. Goldenring and Eunyoung Choi from Vanderbilt University, with 15 and 9 publications, respectively. Jason C. Mills from Baylor College of Medicine followed with 7 publications. The table also reveals the top 10 most-cited authors in this field. Professor Correa Pelayo from Vanderbilt University ranked first with 97 citations, followed by James R. Goldenring with 67 citations and Ki Taek Nam from the Nashville VA Medical Center and the Department of Surgery with 59 citations. An in-depth analysis of author collaboration networks (**Figure 5**) showed that James R. Goldenring and Eunyoung Choi, both from Vanderbilt University, not only published the most papers but also closely collaborated, driving advancements in this field. Both also maintained strong collaboration with Jason C. Mills. Other notable collaborative networks included Professors Marios Giannakis and Jeffrey I. Gordon, as well as Ban Hisayo, Masae Tatematsu, and Tetsuya Tsukamoto, who jointly contributed to advancing this research area.

### 3.4 Analysis of academic journals: publication and citation trends

**Figure 6:**
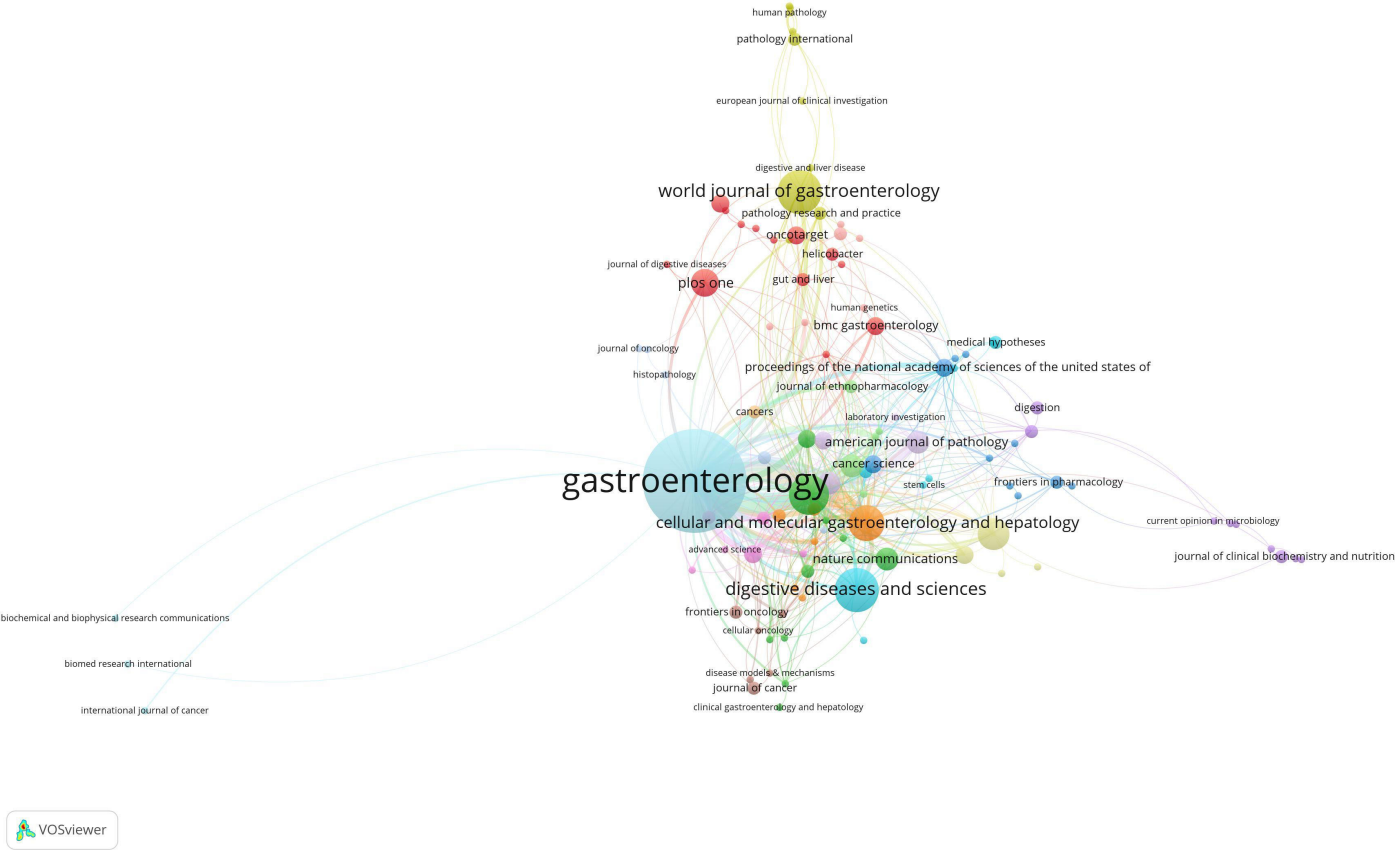
Network map of the issuing journals.

**Figure 7:**
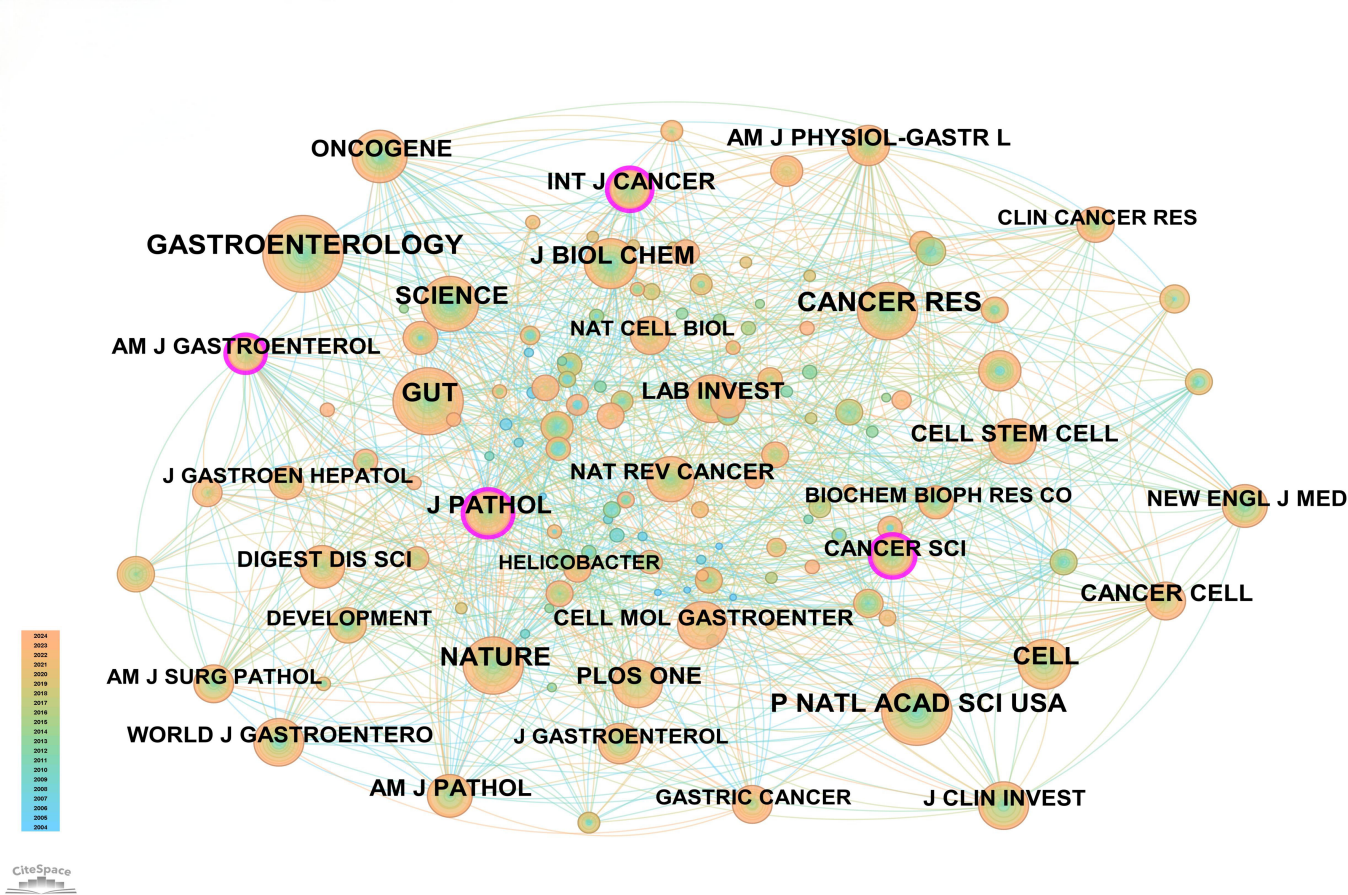
Network map of cited journals.

In this field, 140 academic journals actively participated in publishing related papers. **Figure 6** shows the top 40 journals by publication count. Gastroenterology ranked first with 26 publications, followed by World Journal of Gastroenterology and Digestive Diseases and Sciences, each with 9 publications. Other prominent journals included Gut with 8 publications and Cellular and Molecular Gastroenterology and Hepatology with 7 publications. Notably, among the top 5 journals by publication count, 4 were categorized as Q1 in the Journal Citation Reports (JCR), and one was Q2. Three journals had impact factors exceeding 5, underscoring their high academic influence. In terms of citations, Gastroenterology was not only the journal with the highest publication count but also the most cited, with 188 citations. Other highly cited journals included Cancer Research (151 citations), Gut (148 citations), Nature (145 citations), and PNAS (135 citations). To visually demonstrate the relationships between journals, a co-citation network map was created (**Figure 7**). In the map, node size represents the number of co-citations, and edge thickness reflects the strength of association. Different colors represent the years of cited journals, while the outer purple ring highlights recently prominent journals, providing researchers with intuitive reference information.

### 3.5 In-depth analysis of disciplinary fields

**Figure 8:**
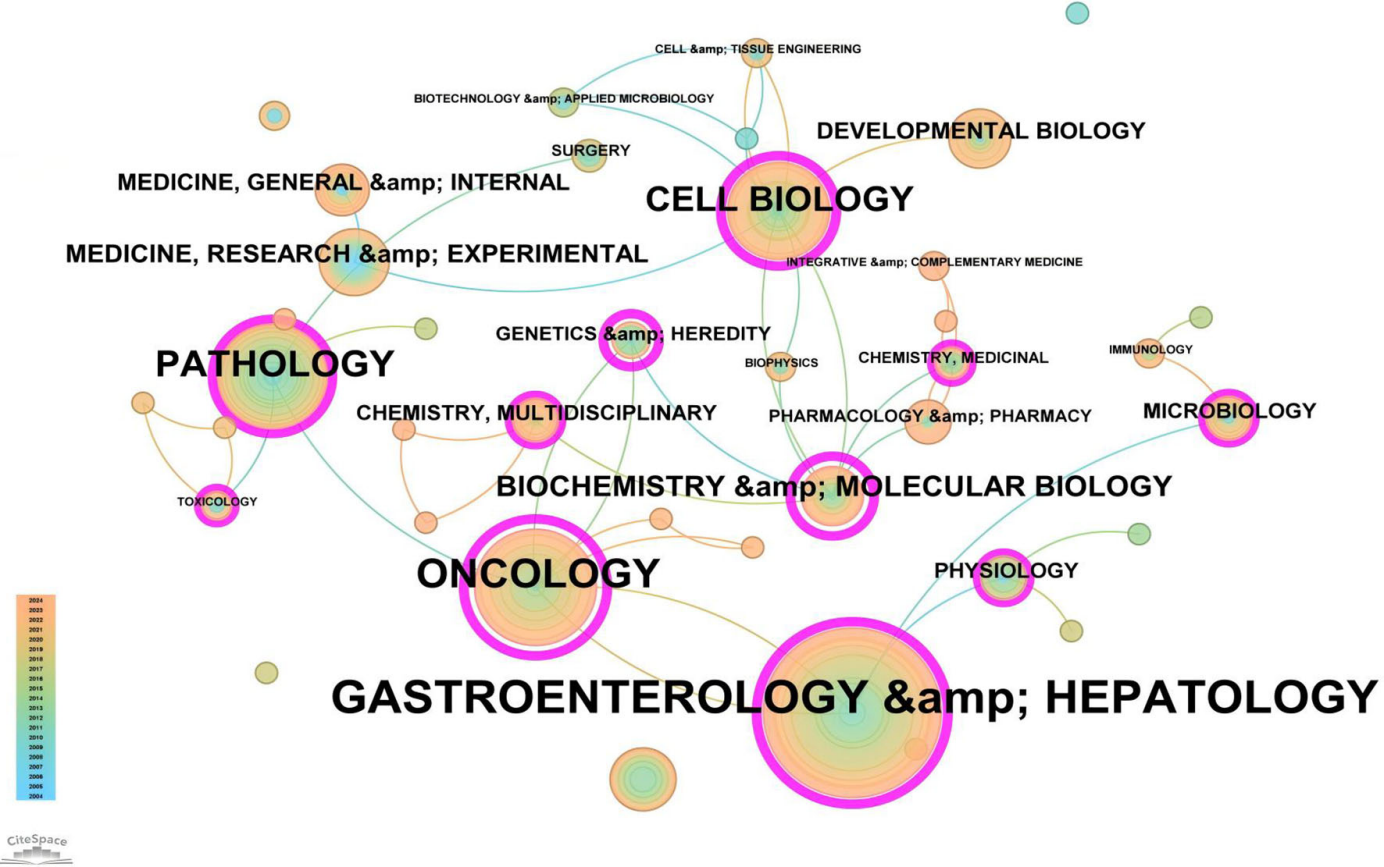
Network map of subject areas for “PLGC-gastric stem cell” and “PLGC-chief cell” studies.

**Figure 9:**
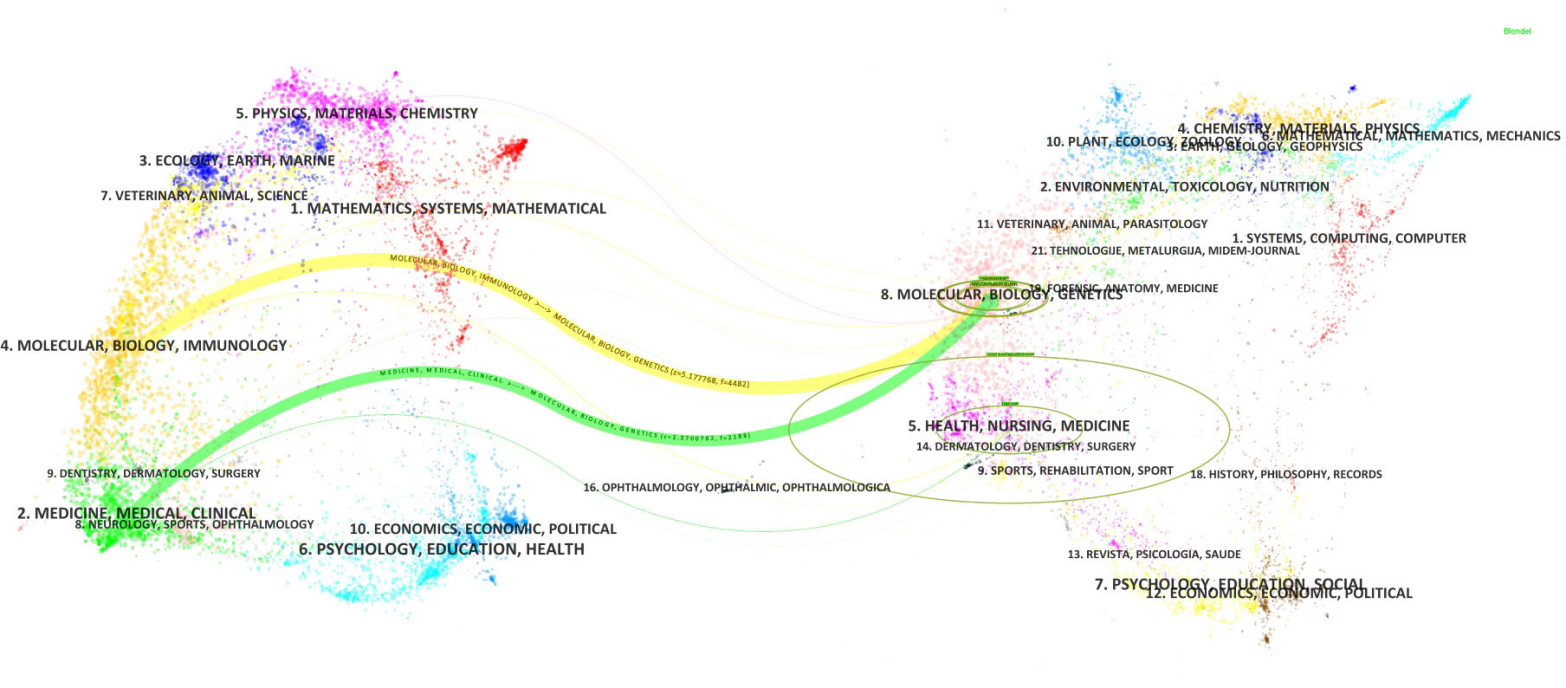
Double image overlay of journals on “PLGC-gastric stem cell” and “PLGC-chief cell” studies.

Based on the classification of 230 papers, a disciplinary field network map was constructed (**Figure 8**). In this visualization, the size of each node reflects the frequency of occurrence for each disciplinary field, while the connections between nodes reveal the degree of correlation and co-occurrence among these fields. Different colors indicate the emergence of disciplinary fields in different years, with the outer purple rings highlighting recent discussions and heightened attention within these areas. Notably, the field of Gastroenterology & Hepatology ranked highest with 86 occurrences, followed by Oncology (49 occurrences) and Pathology (28 occurrences), which together form the core disciplinary cluster of this research area. To further explore the development patterns and trends of these disciplines, a dual-map overlay analysis of journals was employed (**Figure 9**). This method not only illustrates the multidisciplinary influence of research on “PLGC-gastric stem cell” and “PLGC-chief cell,” but also, through visualization, uncovers the intrinsic connections between the knowledge frontier (smaller citing journals on the left) and the knowledge foundation (larger cited journals on the right). The citation links bridging these sides reflect the collaboration intensity between journal categories, with link thickness indicating the level of interaction, thus outlining a clear trajectory of disciplinary dynamics and providing strong support for understanding interdisciplinary relationships. In **Figure 9**, the citing journals predominantly focus on fields such as cluster 2# (Medicine, Medical, Clinical), cluster 4# (Molecular, Biology, Immunology), and cluster 6# (Psychology, Education, Health). Meanwhile, the cited journals are more distributed in cluster 4# (Molecular, Biology, Immunology), cluster 5# (Health, Nursing, Medicine), and cluster 8# (Molecular, Biology, Genetics). Particularly noteworthy is the profound influence of publications in Molecular, Biology, and Immunology (highlighted with yellow trajectories) on the fields of Molecular, Biology, and Genetics. Similarly, papers in Medicine, Medical, and Clinical (green trajectories) are significantly influenced by work in Molecular, Biology, and Genetics, confirming the pivotal role of interdisciplinary integration in driving the advancement of this research domain.

### 3.6 Co-citation analysis of references

**Supplementary Table 3** summarizes the top 10 most-cited publications in the research field of “PLGC-gastric stem cell” and “PLGC-chief cell.” Leading the list is the study titled “Lgr5-expressing chief cells drive epithelial regeneration and cancer in the oxyntic stomach” by Marc Leushacke et al., published in Nature Cell Biology, with 21 citations.

This study focused on the role of Lgr5 in the oxyntic stomach, specifically its expression in gastric body epithelial cells, its function, and its relationship to cancer. It was found that Lgr5+ is selectively expressed in the primary cells at the base of mouse and human gastric body glands. Under normal physiological conditions, these Lgr5+ chief cells do not participate as stem cells in the daily renewal of the gastric body epithelium. However, when the gastric body is injured, Lgr5+ chief cells are activated and transformed into stem cells that drive epithelial regeneration and repair by activating the Wnt signaling pathway. In addition, the study also pointed out that Lgr5+ chief cells are important cells of origin for gastric cancer, and upon activation of oncogenes, they can trigger chemotactic changes in the gastric mucosa and form precancerous lesions. These findings contribute to an in-depth understanding of the homeostatic maintenance and regenerative mechanisms of the gastric body epithelium as well as the process of carcinogenesis, and provide a scientific basis for the development of novel therapeutic strategies against gastric cancer (14).

Following closely is the study by Yoku Hayakawa et al., “Mist1 Expressing Gastric Stem Cells Maintain the Normal and Neoplastic Gastric Epithelium and Are Supported by a Perivascular Stem Cell Niche,” published in Cancer Cell, with 20 citations. This research highlighted the role of Mist1 in the gastric body and found that Mist1-labeled quiescent stem cells, located in the isthmus of the gastric body, are at the origin of all epithelial cell lineages and can serve as cells of origin for gastric cancer. Specifically, Mist1+ stem cells are located in the isthmus of the gastric body and are the stem cells of the gastric body epithelium, with the capacity for self-renewal and multidirectional differentiation. They can differentiate into mucus neck cells, mural cells, surface concave cells, and endocrine cells, etc. Mist1+ stem cells are quiescent under normal conditions but are activated upon injury or inflammatory stimuli to participate in tissue repair and regeneration. In addition, Mist1+ stem cells can serve as cells of origin for gastric cancer, especially in the case of Kras and Apc gene mutations, which can trigger intestinal-type gastric cancer, and in the case of E-cadherin deletion, which can trigger diffuse-type gastric cancer. The study also revealed the role of the Cxcl12/Cxcr4 axis and Wnt5a in the development of gastric cancer, suggesting that these molecules may be potential targets for gastric cancer therapy (15).

Another highly cited study, published by Eunyoung Choi et al. in Gastroenterology and titled “Expression of Activated Ras in Gastric Chief Cells of Mice Leads to the Full Spectrum of Metaplastic Lineage Transitions,” received 19 citations. In this study, it investigated the effect of activated Ras on gastric mucosal chemotaxis by expressing it in mouse gastric master cells. It was found that Mist1-CreERT2Tg/+; LSL-K-Ras(G12D)Tg/+(Mist1-Kras) mice underwent transdifferentiation of gastric master cells after tamoxifen injection to form SPEM, which progressed to IM within 3–4 months. These chemotactic glands expressed markers of SPEM and IM and were infiltrated with macrophages. Lineage tracing confirmed that these chemotactic glands were derived directly from principal cells expressing active Kras. Treatment with the MEK inhibitor selumetinib resulted in IM regression and re-establishment of normal mucosal cells. The study concludes that activation of Ras in gastric master cells can trigger a series of chemotactic lineage transitions, including SPEM and IM. By inhibiting MEK in the Ras signaling pathway, it may be possible to reverse precancerous chemotaxis in the stomach and provide a therapeutic strategy for the prevention of gastric cancer (16). These three groundbreaking studies have not only achieved significant advancements in their respective areas but also laid a solid foundation for subsequent research in the “PLGC-gastric stem cell” and “PLGC-chief cell” field, exerting a profound impact on this domain.

### 3.7 Keyword analysis: co-occurrence, clustering, trends, and timeline evolution

**Figure 10:**
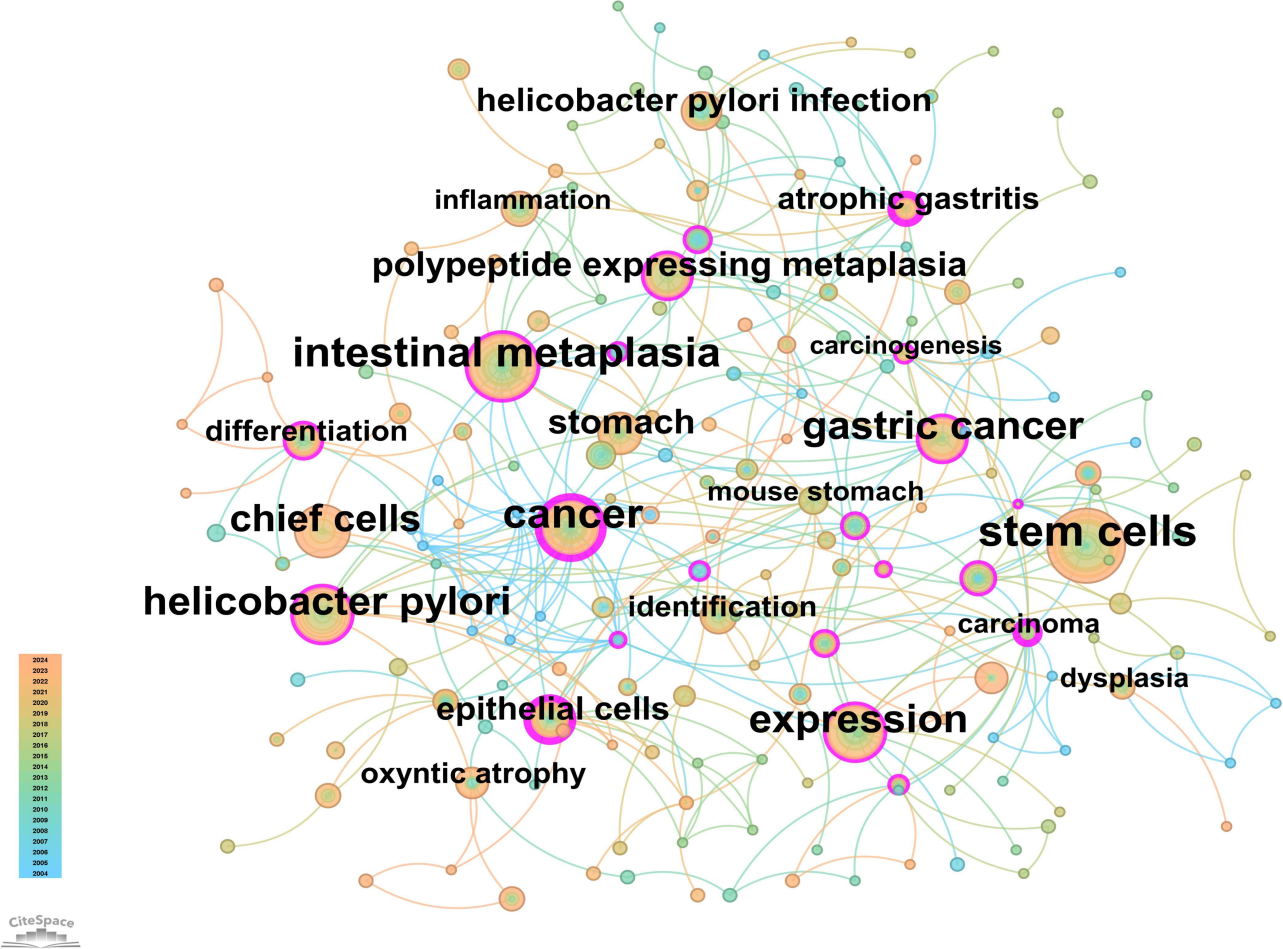
Network map of keywords for “PLGC-gastric stem cell” and “PLGC-chief cell” studies.

**Figure 11:**
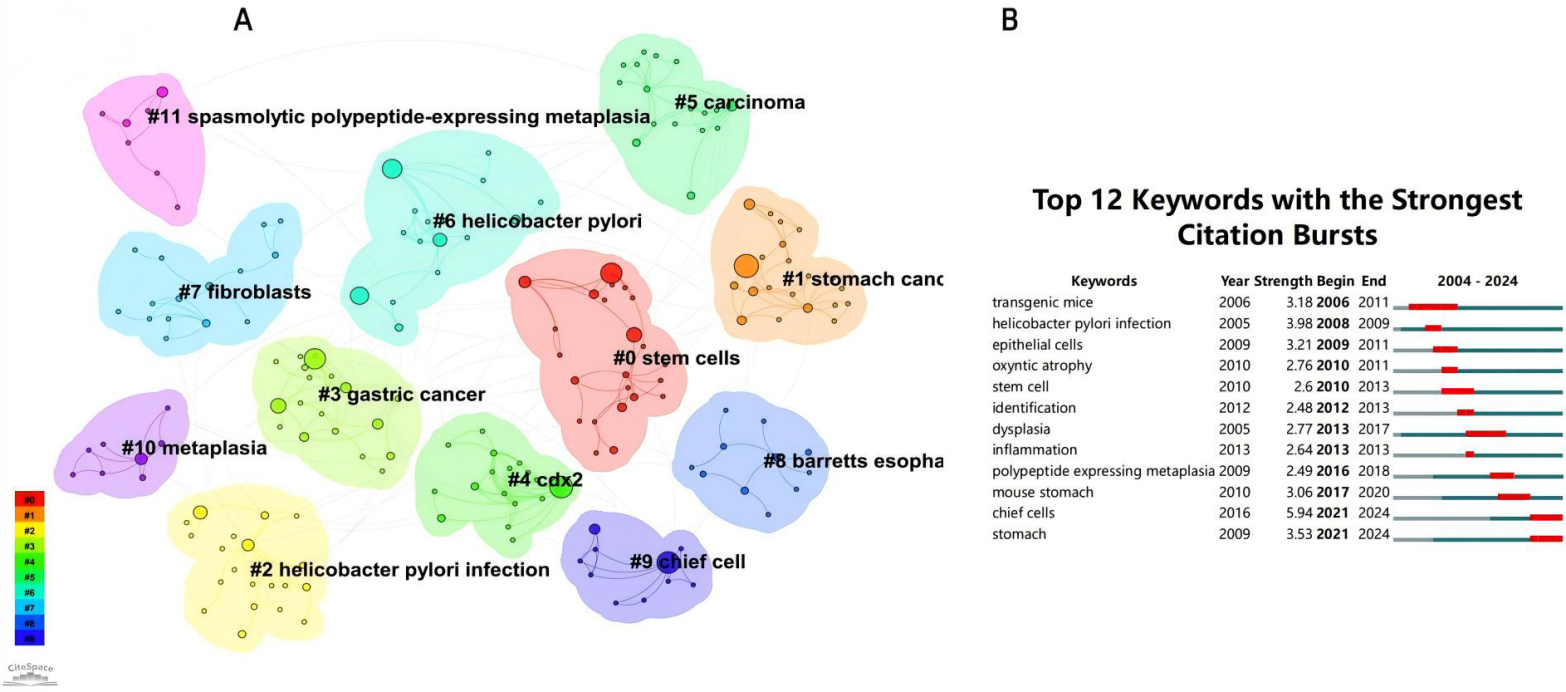
**(A)** Keywords clustering in “PLGC-gastric stem cell” and “PLGC-chief cell” studies. **(B)** The strongest keywords bursts.

From the 230 selected papers, a total of 193 core keywords were extracted. As shown in **Supplementary Table 4**, stem cells, intestinal metaplasia, and cancer emerged as the most frequently occurring keywords. The keyword co-occurrence network (**Figure 10**) vividly illustrates the interconnections among these keywords. The size of the nodes reflects the frequency of keyword occurrence, while the edges between nodes indicate co-occurrence relationships. The color gradient represents the appearance of keywords over time, with purple outer rings highlighting keywords that have gained significant research attention in recent years. To gain a deeper understanding of the thematic distribution in the “PLGC-gastric stem cell” and “PLGC-chief cell” research field, a keyword clustering analysis was conducted. As shown in **Figure 11A**, the keywords were distinctly grouped into nine clusters, encompassing core themes such as stem cells, stomach cancer, H. pylori infection, gastric cancer, Cdx2, carcinoma, fibroblasts, Barrett’s esophagus, chief cell, metaplasia, and spasmolytic polypeptide-expressing metaplasia. Further, a burst detection analysis of keywords revealed their popularity trends and temporal variations (**Figure 11B**). During the early research phase (2004–2010), keywords such as transgenic mice, H. pylori infection, epithelial cells, and oxyntic atrophy showed a notable surge in prominence. In the mid-phase (2010–2016), stem cell, identification, dysplasia, and inflammation emerged as research hotspots. In recent years (2017–2024), keywords like polypeptide expressing metaplasia, mouse stomach, chief cells, and stomach have become focal points, reflecting the field’s evolving research priorities. The cluster analysis reveals distinct thematic groups within the literature, with notable clusters such as “stem cells”, “chief cell”, and “SPEM”, illustrating the interrelatedness of these key topics. The strong citation bursts for keywords such as “oxyntic atrophy” (2010-2011), “stem cell” (2010-2013), and “chief cells” (2021-2024) further indicate a shift in research focus from glandular atrophy to stem cell plasticity in recent years. These findings suggest that emerging research increasingly centers on the molecular mechanisms governing chief cell dedifferentiation and stem cell activation in precancerous gastric lesions.

Based on the keyword clustering, we can derive the potential mechanisms of gastric stem cells and chief cells in the development of gastric precancerous lesions. Firstly, gastric stem cells contribute significantly to the regeneration and repair of the gastric epithelium. When the gastric mucosa is damaged or subjected to chronic stimuli (such as H. pylori infection or excessive gastric acid), gastric stem cells may undergo abnormal proliferation and differentiation, leading to the development of intestinal metaplasia and other precancerous lesions. These lesions are early markers of gastric cancer, and the aberrant proliferation and differentiation of gastric stem cells may serve as an initial trigger for carcinogenesis. Furthermore, chief cells, which are primarily responsible for the secretion of pepsinogen, also play a critical role in gastric cancer precursor lesions. Pepsinogen, when activated to pepsin in the acidic environment of the stomach, is essential for protein digestion. However, the dysregulation of pepsinogen secretion can impact the gastric environment, potentially leading to mucosal damage or other precancerous conditions, such as intestinal metaplasia. Thus, the role of chief cells in precancerous lesions is linked to their ability to contribute to a disrupted gastric microenvironment, which may, in turn, promote carcinogenesis. In summary, gastric stem cells and chief cells have multifaceted roles in gastric precancerous lesions. By influencing the regeneration and differentiation of the gastric epithelium and the stability of the gastric microenvironment, these cells may serve as important early indicators of gastric cancer.

**Figure 12:**
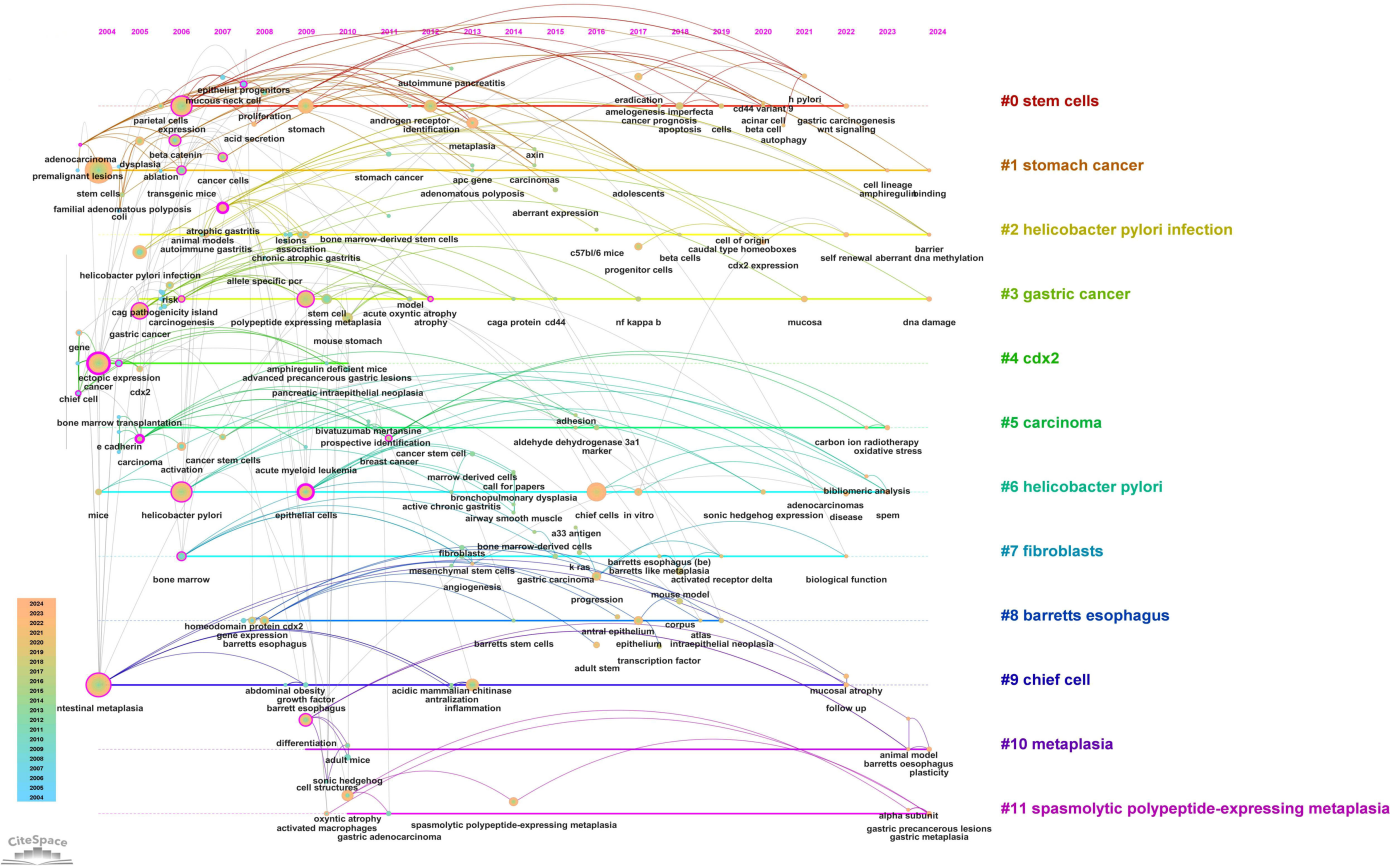
Timeline plot of keywords clustering in “PLGC-gastric stem cell” and “PLGC-chief cell” studies.

**Figure 13:**
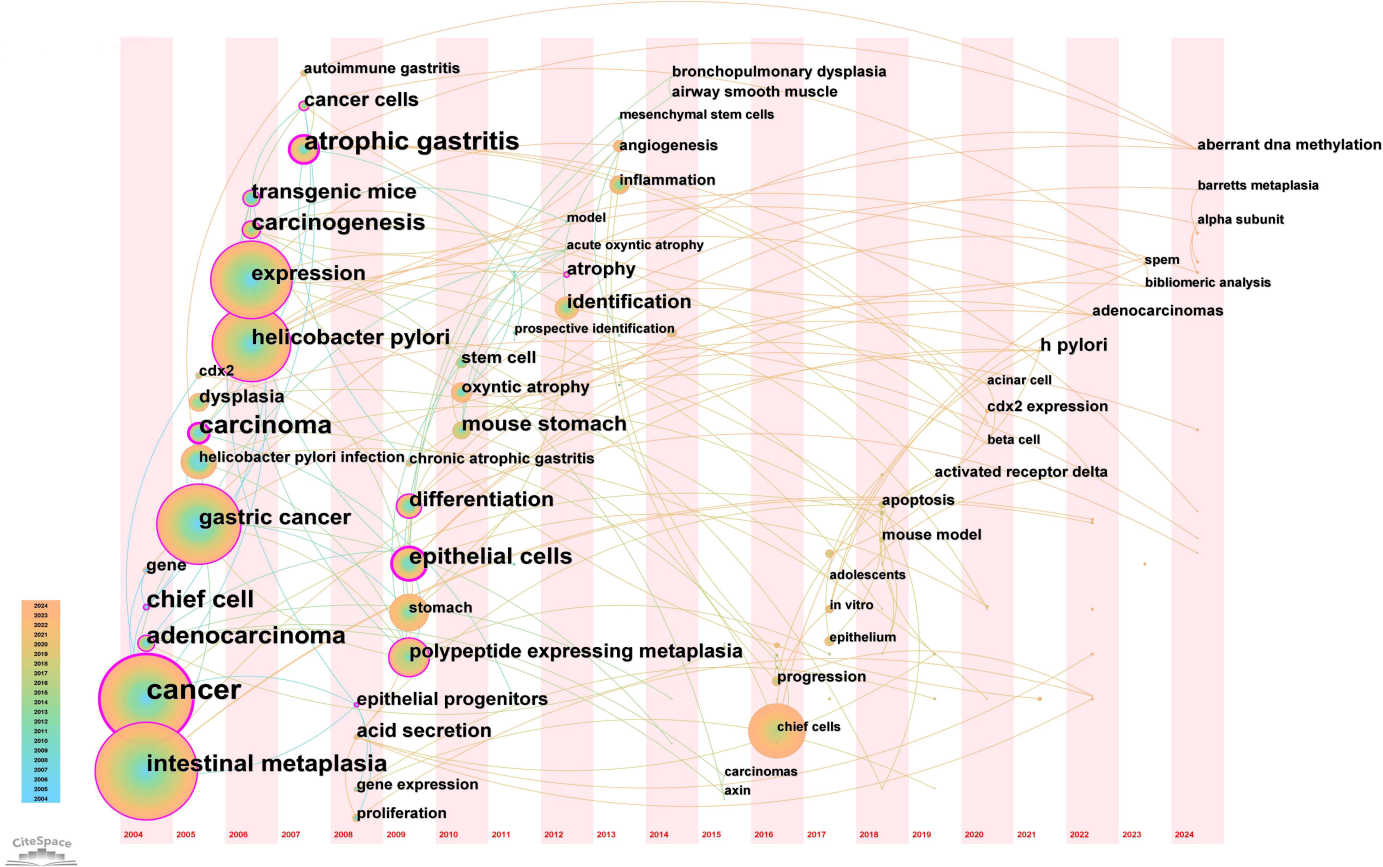
Keyword time zone map in “PLGC-gastric stem cell” and “PLGC-chief cell” studies.

A timeline view of keyword clusters (**Figure 12**) provides an intuitive representation of the temporal evolution of thematic clusters. Within each cluster, nodes are arranged along a single timeline, with node size corresponding to co-citation frequency and position reflecting the chronological order of appearance. Nodes on the left represent earlier, traditional themes, while those on the right indicate emerging research directions. Notably, nodes with purple outer rings mark recent hotspots, hinting at potential future research trends. The timeline visualization demonstrates the progressive refinement of research interests over time. Earlier studies (2004–2010) focused primarily on chronic inflammation (H. pylori infection) and atrophic gastritis, whereas more recent literature (2015–2024) has emphasized cellular lineage tracing and metaplastic transitions (SPEM, CDX2, Lgr5+ stem cells). This transition underscores a paradigm shift from studying inflammation-driven carcinogenesis to investigating stem cell-mediated mucosal remodeling, with significant implications for early detection and therapeutic intervention. Additionally, the keyword timezone map (**Figure 13**) offers a clearer and more intuitive perspective on the annual changes in high-frequency keywords between 2004 and 2024. The connecting lines between keywords in the map reveal the close relationships among keywords across different years, providing robust support for understanding the dynamic development of this research field.

### 3.8 Gene and pathway annotations for “PLGC-gastric stem cell” and “PLGC-chief cell”

**Figure 14:**
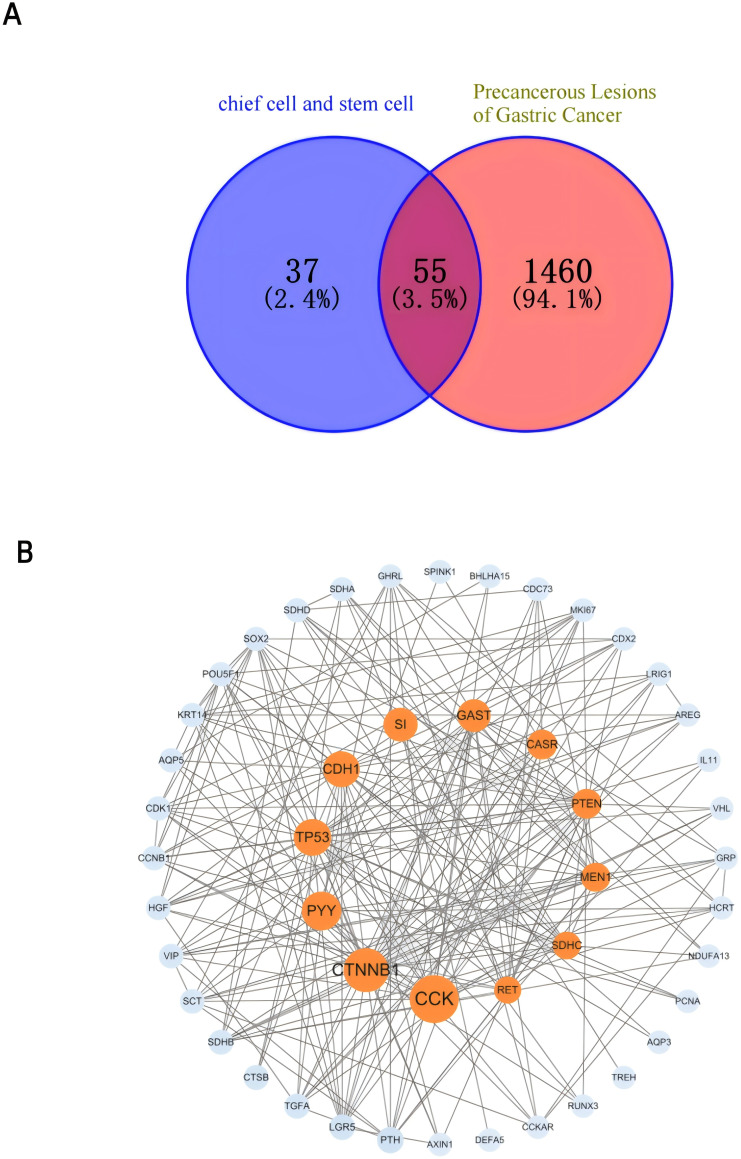
**(A)** Venn diagram of the gastric stem cell, chief cell and PLGC targets. **(B)** Gastric stem cell, chief cell and PLGC target PPI network map.

**Figure 15:**
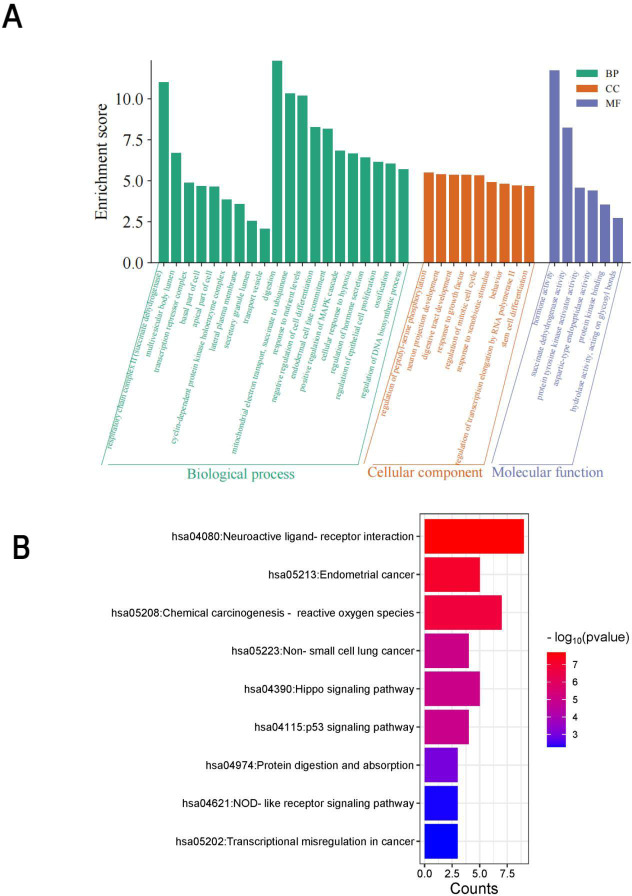
**(A)** Enrichment GO term BP, CC, MF histograms. **(B)** KEGG Bar Chart.

To deepen our understanding of the molecular mechanisms underpinning the “PLGC-gastric stem cell” and “PLGC-chief cell” research domains, we extracted relevant gene-related keywords from the literature. Using the GeneCards database (https://www.genecards.org), we searched for terms such as “precancerous lesions of gastric cancer,” “chronic atrophic gastritis,” “intestinal metaplasia,” “dysplasia,” “intraepithelial neoplasia,” “gastric stem cell,” and “chief cell,” limiting the species to Homo sapiens. This process identified and filtered target genes associated with these domains. Subsequently, the Uniprot database (https://www.uniprot.org/) was used to standardize these targets, ensuring consistency by merging entries and removing duplicates. This yielded 92 targets specifically linked to “gastric stem cell” and “chief cell,” and 1,515 targets related to PLGC-associated conditions. By intersecting these datasets, 55 shared targets were identified and visualized using a Venn diagram (**Figure 14A**). These shared genes were then analyzed using the STRING database to construct a protein-protein interaction (PPI) network with a minimum interaction score threshold of >0.7. The resulting PPI network, visualized with Cytoscape software, represented target proteins as nodes (**Figure 14B**). Based on their degree values, the top 10 hub genes were identified as CCK, CTNNB1, PYY, TP53, CDH1, SI, GAST, CASR, PTEN, and MEN1. To further explore the functional roles of these targets, GO (Gene Ontology) and KEGG (Kyoto Encyclopedia of Genes and Genomes) enrichment analyses were conducted using the Microbial Informatics online platform (https://www.bioinformatics.com.cn). A significance threshold of *P*<0.05 was applied to identify enriched biological functions, and the results were visually represented. GO enrichment analysis revealed that the “GO Biological Processes” category was the most enriched, comprising 20 entries focused on nutrient level responses, negative regulation of cell differentiation, and digestion. The “GO Cellular Components” category included 9 entries, primarily involving the apical and basal parts of the cell, while the “GO Molecular Functions” category contained 6 entries, primarily related to hormone activity and protein kinase binding. Each category was ranked by significance and visually summarized in **Figure 15A**. The KEGG pathway analysis identified nine significantly enriched pathways (*P*<0.05), as shown in **Figure 15B**. These pathways included Neuroactive ligand-receptor interaction, Endometrial cancer, Chemical carcinogenesis - reactive oxygen species, Non-small cell lung cancer, Hippo signaling pathway, p53 signaling pathway, Protein digestion and absorption, NOD-like receptor signaling pathway, and Transcriptional misregulation in cancer.

Based on the GO enrichment and KEGG pathway analyses, several key biological processes and pathways were identified as central to the progression of gastric precancerous lesions. Enriched biological processes, such as signal transduction, response to stress, and regulation of gene expression, reflect the dynamic cellular responses that occur during the transformation of GSCs and chief cells. These processes are particularly important in the context of chronic inflammation and injury, where dysregulated signaling contributes to the development of intestinal metaplasia and dysplasia.

The NF-κB and WNT signaling pathways are key drivers in this transformation, with NF-κB promoting the dedifferentiation of chief cells into SPEM cells, and WNT signaling mediating GSC differentiation into intestinal-type epithelium. These pathways, along with the Hippo signaling pathway, which regulates cell proliferation and apoptosis, are critical in maintaining the balance between normal tissue regeneration and pathological transformation. The p53 pathway, recognized as a guardian of the genome, is also central to the prevention of carcinogenesis. Dysregulation of p53 in GSCs and chief cells leads to genomic instability, facilitating the progression from metaplasia to dysplasia. Additionally, the enrichment of pathways such as reactive oxygen species (ROS) production and transcriptional misregulation in cancer underscores the importance of oxidative stress and abnormal gene regulation in gastric carcinogenesis.”

By linking these pathways with GSC and chief cell behaviors, these findings not only highlight critical molecular mechanisms but also suggest potential therapeutic targets for preventing the progression of gastric precancerous lesions. Future research should focus on elucidating the precise roles of these signaling pathways and their interactions in PLGC.

## 4 Discussion

### 4.1 Overview

In the gastric epithelium, GSCs and chief cells are vital for maintaining tissue homeostasis and facilitating repair after injury, though they differ significantly in function, anatomical location, and cellular characteristics. GSCs, a heterogeneous population of regenerative cells, are responsible for the continuous renewal and regeneration of the gastric epithelium. These cells are broadly classified into isthmic stem cells (17), located in the gastric gland isthmus and expressing the marker Lgr5, and corpus stem cells, found in the gastric corpus and marked by Troy. Isthmic stem cells are highly proliferative, capable of differentiating into all major epithelial cell types, including parietal cells, mucous cells, and enteroendocrine cells, playing a crucial role in maintaining epithelial turnover and glandular homeostasis. In contrast, corpus stem cells remain quiescent under normal conditions but become activated during stress or injury, contributing to epithelial repair (18). Although their differentiation potential is more restricted compared to isthmic stem cells, corpus stem cells can replenish specific cell types, such as chief cells and parietal cells, when necessary. Chief cells, located at the base of gastric glands, are specialized for the production and secretion of pepsinogen, the precursor of the enzyme pepsin, essential for protein digestion. In their differentiated state, these cells also produce other molecules involved in digestion, such as lipases and intrinsic factor, highlighting their importance in gastric function. Despite their long lifespan, chief cells are continually renewed by GSCs. Under pathological conditions, such as chronic injury or inflammation, chief cells exhibit remarkable plasticity by dedifferentiating into a progenitor-like state, leading to the formation of SPEM cells. SPEM cells are often associated with gastric metaplasia and may serve as precursors to gastric cancer (19). Their coexistence with intestinal metaplasia in the gastric mucosa underscores the adaptive response of chief cells to sustained damage or stress. In summary, GSCs and chief cells represent two integral components of the gastric epithelium, with distinct roles in maintaining tissue integrity and responding to injury. While GSCs, particularly Lgr5+ and Troy+ cells, ensure continuous epithelial renewal (8), chief cells play a critical role in digestion and exhibit dynamic adaptability under pathological conditions, contributing to gastric metaplasia. Understanding the interplay between these cell types is crucial for elucidating their roles in gastric diseases and exploring potential therapeutic interventions.

An analysis of the WoSCC database from 2004 to 2024 in the field of “PLGC-gastric stem cell” and “PLGC-chief cell” reveals 230 publications authored by 151 researchers from 156 organizations across 31 countries. Over the past two decades, the publication volume has shown a steady growth trend. The top three countries in terms of publication output are the United States, China, and Japan. Close collaboration is evident among countries, with Canada having the highest centrality score of 0.39, indicating its bridging role in international cooperation and scholarly exchange. Among the 156 institutions, Vanderbilt University and WUSTL, both located in the United States, are the leading contributors in terms of publication volume. Eight of the top ten institutions are based in the United States, reflecting the country’s significant attention to research on gastric stem cells, chief cells, and precancerous gastric lesions, and highlighting its prominent position and contribution to this field. Professor James R. Goldenring of Vanderbilt University is the most prolific author, having initiated related research on gastric stem cells in 2006. Over 20 years, he has published 15 papers in this domain. His most recent 2024 study identifies isthmic progenitor cells as tissue-specific stem cells in the gastric body, which maintain mucosal homeostasis by balancing proliferation and differentiation into gastric epithelial lineages. Progenitor cells, intermediate between stem cells and mature cells, possess differentiation potential. These cells originate from stem cells and further differentiate into terminally differentiated cells such as chief cells and parietal cells. During mucosal injury, progenitor cells become rapidly activated. Post-injury mucosal regeneration drives isthmic progenitor cells to transition into surface cell lineages (20). Amphiregulin (AREG) treatment facilitates the repopulation of surface cells while suppressing progenitor cell commitment to surface differentiation. In contrast, transforming growth factor-α does not alter surface cell regeneration but induces expansion of the surface cell population. Research by Prof. James R. Goldenring’s team has shown that the presence of dual-positive telocytes, characterized by FOXL1 and PDGFRα expression, within the gastric isthmus area (21). A study by Professor James R. Goldring’s team showed the presence of double-positive telocytes in the region of the gastric isthmus, which are characterized by the expression of FOXL1 and PDGFRα. The number of telocytes increased significantly with the progression of metaplasia. Their spatial distribution extended beyond the confines of the isthmus, spreading throughout the glandular structure and closely mirroring the proliferation zone’s expansion. This redistribution of telocytes was not a nonspecific reaction to mucosal injury but was instead linked to the formation of a niche for metaplastic cells at the gland’s base. Lineage tracing studies further revealed the dynamic recruitment of telocytes to these newly formed metaplastic cell niches. Concurrently, the study detected the expression of Wnt5a, Bmp4, and Bmp7 within the PDGFRα-expressing telocytes, suggesting their active involvement in the metaplastic process. study validated the presence of dual-positive telocytes, characterized by FOXL1 and PDGFRα expression, within the gastric isthmus area (22).

Research on “PLGC-gastric stem cell” and “PLGC-chief cell” are currently concentrated in disciplines such as molecular biology, immunology, medicine, and clinical science. In certain cases, part of the knowledge base comprises references co-cited by scholars involved in relevant studies. Among the three most frequently co-cited references, two emphasize the role of chief cells in gastric cancer progression, identifying them as the direct origin of metaplastic cellular lesions. These studies define Lgr5+ chief cells as the primary initiating cells of gastric cancer and highlight the critical role of Lgr5+ cells in maintaining the homeostatic stem cell pool (14). The third reference focuses on quiescent Mist1+ gastric stem cells in the gastric isthmus as the origin of all epithelial lineages. These cells can serve as the cellular origin for all histological types of gastric cancer (15). Additionally, the study identifies the Cxcl12/Cxcr4 axis, comprising endothelial cells and innate lymphoid cells (ILCs), as a regulator of normal and tumor-associated gastric stem cell niches (16).

### 4.2 Research hotspots and emerging trends

In the era of information overflow and rapidly advancing technology, staying abreast of the latest developments in a research field is paramount for scientists. Bibliometric analysis, particularly through the co-occurrence of keywords, serves as an effective tool to unveil the central focuses of specific research domains (23). By integrating time-series analysis with visualization techniques, the trajectories of emerging hotspots can be clearly delineated. Furthermore, analyzing clustering patterns in references and identifying citation surges enables the identification of nascent topics within a discipline. This study employed multiple methodologies, including keyword co-occurrence analysis, timeline evolution mapping, citation burst detection, KEGG pathway mapping, and GO functional annotation. These approaches comprehensively evaluated the research hotspots and advancements in the fields of “PLGC-gastric stem cell” and “PLGC-chief cell.” The following sections present an integrated discussion and analysis of 6 core hotspots and emerging trends within these domains.

#### 4.2.1 Roles of GSCs and chief cells in normal gastric homeostasis

Gastric homeostasis depends on the coordinated functions of GSCs and chief cells, which fulfill distinct yet complementary roles. GSCs, situated primarily in the isthmus and base of gastric glands, are characterized by their ability to self-renew and differentiate into multiple epithelial cell lineages, including mucous cells, parietal cells, chief cells, and enteroendocrine cells. This regenerative capacity ensures the structural and functional integrity of the gastric mucosa. GSC populations include actively cycling Lgr5-expressing cells at the gland base and quiescent Troy-expressing cells in the corpus region, forming a hierarchical system that allows adaptive responses under normal and injury conditions (8). Chief cells, located at the base of the gastric glands, are key players in digestive function through their secretion of pepsinogen, the precursor to pepsin, essential for protein digestion. Beyond their traditional role, chief cells exhibit remarkable plasticity under acute injury, dedifferentiating into progenitor-like states to aid epithelial repair. This dedifferentiation provides an auxiliary mechanism for regeneration, compensating for compromised GSC function. The interplay between GSCs’ regenerative properties and the plasticity of chief cells establishes a robust system for maintaining gastric gland function under physiological and pathological conditions.

#### 4.2.2 Differing roles of stem cells and chief cells in precancerous lesions

The progression of gastric precancerous lesions, including CAG, IM, and Dys, involves distinct but interconnected contributions from GSCs and chief cells.

##### 4.2.2.1 Roles of stem cells and chief cells in CAG

During CAG, persistent inflammatory signaling, primarily through IL-1β and NF-κB activation, disrupts the regenerative potential of GSCs. This impairment leads to epithelial thinning and glandular architectural remodeling. Concurrently, mature chief cells exhibit plasticity by undergoing either apoptosis or transdifferentiation into SPEM cells. This adaptive process, though initially reparative, establishes a permissive environment for further metaplastic and dysplastic progression.

##### 4.2.2.2 Roles of stem cells and chief cells in IM

Persistent inflammatory signaling, particularly through H. pylori-mediated activation of NF-κB and WNT pathways (24, 25), alters GSC fate decisions, shifting differentiation from normal gastric epithelial cells to intestinal-type epithelial cells. This lineage conversion contributes to the characteristic glandular remodeling seen in IM. Concurrently, chief cell-derived SPEM cells frequently emerge alongside IM as part of an adaptive response to chronic injury. SPEM cells exhibit increased plasticity and may provide a transient regenerative niche; however, they also serve as a potential precursor to dysplastic transformations. The co-occurrence of IM and SPEM highlights a crucial crossroad in the evolution of gastric precancerous lesions, with GSCs and chief cells undergoing distinct yet coordinated changes.

##### 4.2.2.3 Roles of stem cells and chief cells in dysplasia

The transition from metaplasia to dysplasia involves a further deviation of GSCs from normal differentiation programs, driven by the hyperactivation of oncogenic pathways such as WNT, YAP, and Notch (26). Dysregulated GSC proliferation results in epithelial architectural distortion, nuclear atypia, and loss of glandular polarity, all hallmark features of dysplasia. While chief cells play a less direct role at this stage, persistent SPEM cells may contribute to the dysplastic microenvironment by secreting inflammatory mediators and interacting with stromal components that promote neoplastic progression. The interplay between GSC-driven dysplastic expansion and the residual presence of metaplastic chief cells suggests a dynamic and evolving landscape in gastric carcinogenesis.

Recent studies have highlighted a controversy surrounding the cellular origin of SPEM. While lineage-tracing experiments in murine models(Mist1-CreERT2, Rosa-LSL-YFP) have demonstrated that mature chief cells can dedifferentiate into SPEM under acute injury (16), other investigations propose that isthmic stem cells, upon aberrant activation by chronic inflammatory signaling, may also give rise to SPEM-like lineages (15). These divergent views likely stem from differences in model systems, injury paradigms, and marker specificity. Resolving this controversy will require refined spatial-temporal lineage tracing and the integration of single-cell transcriptomic profiling.

In addition, regarding the key signaling pathways (NF-κB, WNT, and YAP) we further identified their stage-specific roles in the progression of gastric precancerous lesions. During the early phase of chronic atrophic gastritis, persistent activation of the NF-κB pathway sustains a pro-inflammatory microenvironment that disrupts the homeostatic differentiation of GSCs and contributes to mucosal injury. In the stage of intestinal metaplasia, upregulation of the WNT pathway plays a pivotal role in directing GSC differentiation toward an intestinal epithelial phenotype, while also facilitating the dedifferentiation of chief cells into SPEM. In the dysplastic phase, aberrant activation of the YAP pathway, often in synergy with WNT signaling, promotes uncontrolled GSC proliferation, inhibits terminal differentiation, and drives architectural disorganization characteristic of neoplastic transformation. Together, these signaling cascades orchestrate the pathological remodeling of the gastric mucosa through their dynamic regulation of GSC and chief cell plasticity, thereby contributing to the multistep progression toward malignancy.

These stage-specific cellular dynamics underscore the pivotal roles of both GSCs and chief cells in the development of gastric precancerous lesions. By clarifying their distinct contributions and spatiotemporal transitions, we provide new insights into potential therapeutic interventions aimed at modulating metaplastic and dysplastic progression.

#### 4.2.3 Biomarkers for GSCs and chief cells

Gastric stem cells are identified in various regions of the stomach, with distinct populations exhibiting unique marker expressions. In the antrum, stem cells are marked by Lgr5+ and AQP5+, which are involved in the bidirectional proliferation and differentiation of gastric units. The corpus region reveals a more complex landscape, with TFF2 mRNA, Mist1+ cells (27), and Troy+ mature chief cells identified as potential stem cell candidates. Additionally, Sox2, eR1, Lrig1, Sox9 (28) and Bmi1 are markers associated with gastric stem cells in both the antrum and corpus, indicating their role in tissue regeneration (7). Chief cells, known for their role in pepsinogen secretion, have also been recognized for their stem cell-like properties. Mist1, a marker traditionally associated with chief cells, has been implicated in the maintenance of these cells and their potential to act as progenitors in regeneration processes. Lgr5 is another marker found in a subset of chief cells, suggesting their involvement in tissue renewal. The plasticity of chief cells is further supported by the expression of Troy, which, along with Mist1 and other markers, indicates their capacity to act as reserve stem cells in response to injury (29). Understanding the cellular and molecular underpinnings of gastric stem cells and chief cells is not only essential for elucidating the physiological processes of gastric epithelial maintenance but also for deciphering the pathogenesis of gastric precancerous lesions and cancer. The identification of these cells and their markers provides a foundation for developing targeted therapies and preventive strategies against gastric diseases.

#### 4.2.4 Factors influencing variability in research observations

Variations in research findings related to GSCs and chief cells can often be attributed to differences in experimental models and the molecular markers or pathways studied. Acute injury models typically emphasize chief cell dedifferentiation, demonstrating the formation of SPEM cells as a repair mechanism. In contrast, chronic inflammation models, such as those simulating H. pylori infection, highlight the role of GSCs in driving metaplastic and dysplastic transitions under sustained inflammatory signaling. Furthermore, the choice of molecular markers influences observations; for instance, the cell cycle inhibitor p57 regulates chief cell dedifferentiation during injury (29), whereas WNT and NF-κB signaling pathways primarily impact GSC proliferation and differentiation toward intestinal lineages during metaplasia. These experimental variables reflect differing focuses among studies and underscore the need to contextualize findings within specific models (24). A comprehensive understanding of these factors is essential for integrating diverse observations and developing targeted strategies to address gastric precancerous lesions.

#### 4.2.5 Proposed unified model: coordinated roles of chief cells and stem cells in precancerous lesions

A unified model that integrates the spatial and temporal dynamics of chief cells and GSCs provides valuable insights into their coordinated roles in the progression of gastric precancerous lesions. The chief cells located at the base of the gland act as first responders during acute injury, and the chief cells exhibit reduced autophagy, formation and proliferation of mucus granules, and increased levels of ROS and apoptosis, which are involved in epithelial repair and maintenance of tissue homeostasis through de-differentiation to form SPEM cells (30). Under chronic inflammatory conditions, such as prolonged H. pylori infection, GSCs in the isthmus become the predominant drivers of abnormal proliferation and differentiation, leading to intestinal metaplasia and dysplasia. Chief cells, confined to the gland base, primarily dedifferentiate into SPEM cells, creating a reparative niche but potentially contributing to metaplasia under persistent stress. GSCs, with their broader differentiation potential, respond to chronic signaling dysregulation, driven by WNT and NF-κB pathways, to generate intestinal metaplasia. Continued aberrant signaling fosters hyperproliferation and the emergence of dysplastic cells, marking the progression to precancerous conditions. This model underscores the dynamic interplay between chief cells and GSCs during lesion progression and highlights their complementary roles in injury response and disease evolution.

#### 4.2.6 Therapeutic implications and future directions

Understanding the roles of GSCs and chief cells in precancerous lesions opens promising avenues for early therapeutic intervention. Dysregulated pathways like WNT and YAP are critical drivers of GSC proliferation and abnormal differentiation during chronic inflammation. Targeted modulation of these pathways using small molecule inhibitors or biologics could restore normal GSC function, preventing the progression of intestinal metaplasia and dysplasia. Similarly, preserving chief cell integrity is essential for mitigating acute injury. Molecular regulators such as p57 control chief cell dedifferentiation, and their modulation could enhance repair while preventing maladaptive transformations into SPEM cells under chronic conditions (29). In combination with relevant clinical studies, the abnormal expression of Ki67 and CD44v9 marker proteins in SPEM cells indicates problems with cell proliferation. We should aim to influence the proliferation and differentiation functions of SPEM cells by regulating cellular energy metabolism, ribosome function, cell communication, and apoptotic signaling pathways, effectively inhibiting the abnormal expression of Ki67 and CD44v9 marker proteins in these cells (31). Additionally, the deletion of the CHIA gene in principal cells leads to the expansion of the endoplasmic reticulum, upregulation of pyroptosis and endoplasmic reticulum stress markers, indicating chief cell pyroptosis. The pyroptosome NLRP3 induces the release of the alarmin IL-33, mediating M2 macrophage polarization and promoting SPEM (19). Therefore, we should explore therapeutic targets by improving principal cell pyroptosis and immune regulation.

Future research should prioritize comparative studies across experimental models to validate the temporal and spatial dynamics of these cells under various injury and inflammatory conditions. Although bibliometric data underscores the growing attention to SPEM and chief cells, few studies have explored the molecular drivers of their transition to metaplasia. Future work should prioritize the longitudinal spatial mapping of GSC and chief cell niches during PLGC progression, utilizing single-cell sequencing to identify biomarkers of specific stages. Additionally, identifying early-stage biomarkers for cellular changes could enable timely interventions. A deeper understanding of the interplay between GSCs and chief cells will not only inform novel therapeutic approaches but also support the prevention and early detection of gastric cancer.

Potential candidates include Lgr5+ stem cells, which play a role in glandular regeneration and may indicate early dysregulated proliferation; Mist1, a key regulator of chief cell plasticity and SPEM formation; and CD44v9, which has been linked to oxidative stress resistance in early-stage gastric neoplasia. These markers could serve as targets for diagnostic and risk stratification strategies.

In terms of therapeutic translation, signaling pathways such as WNT, NF-κB and YAP present promising avenues for clinical intervention. The WNT pathway is central to GSC self-renewal and differentiation, and modulating its activity may offer strategies to reverse metaplastic transitions. The NF-κB pathway, a key mediator of chronic inflammation, remains a critical target for preventing sustained epithelial damage. Meanwhile, YAP signaling plays an emerging role in promoting cellular proliferation and plasticity in dysplastic progression, suggesting that targeted inhibitors could mitigate its oncogenic effects.

Given China’s disproportionately high gastric cancer burden—accounting for nearly 40% of global cases—regional research strategies have evolved distinct characteristics. In China and Japan, there is a strong emphasis on integrative medicine approaches, particularly the use of traditional Chinese medicine for early intervention in precancerous lesions. For instance, traditional Chinese medicine compounds are often explored for their capacity to modulate WNT, NF-κB, oxidative stress and immune regulation pathways in gastric epithelium (31, 32). In contrast, research from Europe and North America focuses more on genetic predisposition, stem cell-niche interactions, and immunomodulation. These differences not only reflect divergent medical paradigms but also underscore the value of regionalized prevention and therapeutic strategies. Future bibliometric analyses may benefit from stratifying outputs by regional research focus and clinical application.

### 4.3 Advantages and constraints

This study represents the first bibliometric analysis of research on “PLGC-gastric stem cell” and “PLGC-chief cell” over the past two decades, offering an impartial perspective on evolving trends and research priorities. Using multi-dimensional bibliometric tools, the analysis provides a comprehensive understanding of research developments. However, limitations include reliance on the WoSCC database, which may exclude relevant data from other sources. Additionally, excluding non-English publications and non-article formats might introduce some bias, though the impact is minimal given the extensive coverage of WoSCC. Discrepancies among bibliometric tools and inherent algorithmic limitations may also obscure the contributions of emerging researchers. Despite these constraints, the study provides a robust framework for understanding key trends in this research domain.

### 4.4 Summary

The field of “PLGC-gastric stem cell” and “PLGC-chief cell” is advancing rapidly, with significant implications for gastric cancer prevention and secondary interventions. Early therapeutic strategies targeting abnormal GSC proliferation and differentiation could reverse the progression of PLGC to gastric cancer. The discovery of chief cell dedifferentiation into SPEM cells as a precursor to PLGC further underscores their importance in disease progression. Enhanced international collaboration and future research focusing on these cell types will provide new insights and directions for combating gastric precancerous lesions and improving patient outcomes.

Future research should prioritize integrating single-cell sequencing and spatial transcriptomics to delineate the precise lineage hierarchies of gastric stem and chief cells. Additionally, exploring epigenetic regulation in metaplastic transformation may yield novel targets for therapeutic intervention. By combining these insights, the field can move closer to developing precision medicine approaches for gastric cancer prevention and treatment.

## Data Availability

The datasets presented in this study can be found in online repositories. The names of the repository/repositories and accession number(s) can be found in the article/**Supplementary Material**.
