# Supplementary material for: Dynamic role of gastric stem cells and chief cells in precancerous lesions of gastric cancer: global knowledge mapping and emerging trends based on bibliometric analysis from 2004 to 2024
Source: Front Oncol. 2025 May 16;15:1556009. doi: 10.3389/fonc.2025.1556009 (PMC12122518; doi:10.3389/fonc.2025.1556009)
Supplement: Supplementary file 1 [file Table1.docx]

Table1 The top 10 countries/regions and institutions for “PLGC-gastric stem cell” and “PLGC-chief cell” publications.

| Country | Count | Centrality | Institution | Count | Centrality |
| --- | --- | --- | --- | --- | --- |
| USA | 98 | 0 | Vanderbilt University | 21 | 0.24 |
| PEOPLES R CHINA | 62 | 0 | Washington University | 18 | 0.16 |
| JAPAN | 50 | 0.18 | US Department of Veterans Affairs | 16 | 0.01 |
| SOUTH KOREA | 18 | 0.25 | Veterans Health Administration | 16 | 0.01 |
| GERMANY | 13 | 0.38 | Columbia University | 14 | 0.06 |
| ENGLAND | 10 | 0.34 | Harvard University | 11 | 0.02 |
| SWEDEN | 8 | 0.23 | University of Tokyo | 10 | 0.04 |
| CANADA | 7 | 0.39 | VA Tennessee Valley Healthcare System | 10 | 0 |
| ITALY | 6 | 0 | Harvard Medical School | 8 | 0.03 |
| SINGAPORE | 6 | 0.09 | Seoul National University | 8 | 0.05 |
