# Supplementary material for: Dynamic role of gastric stem cells and chief cells in precancerous lesions of gastric cancer: global knowledge mapping and emerging trends based on bibliometric analysis from 2004 to 2024
Source: Front Oncol. 2025 May 16;15:1556009. doi: 10.3389/fonc.2025.1556009 (PMC12122518; doi:10.3389/fonc.2025.1556009)
Supplement: Supplementary file 2 [file Table2.docx]

Table2 The top 10 most prolific and cited authors in the field of “PLGC-gastric stem cell” and “PLGC-chief cell”.

| Author | Count | Cited Author | Count |
| --- | --- | --- | --- |
| Goldenring, James R | 15 | CORREA P | 97 |
| Choi, Eunyoung | 9 | GOLDENRING JR | 67 |
| Mills, Jason C | 7 | NAM KT | 59 |
| Wang, Timothy C | 4 | BARKER N | 46 |
| Hayakawa, Yoku | 3 | CHOI E | 38 |
| Li, Taolang | 2 | HAYAKAWA Y | 32 |
| Han, Young Min | 2 | FOX JG | 25 |
| Konieczny, Stephen F | 2 | HOUGHTON J | 23 |
| Ban, Hisayo | 2 | NOMURA S | 23 |
| Caldwell, Brianna | 2 | PETERSEN CP | 21 |
