# Supplementary material for: Dynamic role of gastric stem cells and chief cells in precancerous lesions of gastric cancer: global knowledge mapping and emerging trends based on bibliometric analysis from 2004 to 2024
Source: Front Oncol. 2025 May 16;15:1556009. doi: 10.3389/fonc.2025.1556009 (PMC12122518; doi:10.3389/fonc.2025.1556009)
Supplement: Supplementary file 3 [file Table3.docx]

Table 3 The top 10 most cited articles in “PLGC-gastric stem cell” and “PLGC-chief cell” studies.

|  | | Title | First Author | DOI | Journal | Year | Citations |
| --- | --- | --- | --- | --- | --- | --- | --- |
| 1 | Lgr5-expressing chief cells drive epithelial regeneration and cancer in the oxyntic stomach | Marc Leushacke | 10.1038/ncb3541 | Nature Cell Biology | 2017 | 21 |  |
| 2 | Mist1 Expressing Gastric Stem Cells Maintain the Normal and Neoplastic Gastric Epithelium and Are Supported by a Perivascular Stem Cell Niche | Yoku Hayakawa | 10.1016/j.ccell.2015.10.003 | Cancer Cell | 2015 | 20 |  |
| 3 | Expression of Activated Ras in Gastric Chief Cells of Mice Leads to the Full Spectrum of Metaplastic Lineage Transition | Eunyoung Choi | 10.1053/j.gastro.2015.11.049 | Gastroenterology | 16 | 19 |  |
| 4 | Lgr5(+ve) stem cells drive self-renewal in the stomach and build long-lived gastric units in vitro | Nick Barker | 10.1016/j.stem.2009.11.013 | Cell Stem Cell | 2010 | 16 |  |
| 5 | Comprehensive molecular characterization of gastric adenocarcinoma | Bass AJ | 10.1038/nature13480 | Nature | 2014 | 13 |  |
| 6 | Differentiated Troy+ chief cells act as reserve stem cells to generate all lineages of the stomach epithelium | Daniel E Stange | 10.1016/j.cell.2013.09.008 | Cell | 2013 | 12 |  |
| 7 | Single-Cell Transcriptional Analyses Identify Lineage-Specific Epithelial Responses to Inflammation and Metaplastic Development in the Gastric Corpus | Kevin A Bockerstett | 10.1053/j.gastro.2020.08.027 | Gastroenterology | 2020 | 12 |  |
| 8 | Cancer incidence and mortality worldwide: sources, methods and major patterns in GLOBOCAN 2012 | Jacques Ferlay | 10.1002/ijc.29210 | VA Tennessee Valley Healthcare System | 2015 | 11 |  |
| 9 | Gastric cancer originating from bone marrow-derived cells | Jeanmarie Houghton | 10.1126/science.1099513 | Science | 2004 | 11 |  |
| 10 | Macrophages promote progression of spasmolytic polypeptide-expressing metaplasia after acute loss of parietal cells | Christine P Petersen | 10.1053/j.gastro.2014.02.007 | Gastroenterology | 2014 | 10 |  |
