# Supplementary material for: Dynamic role of gastric stem cells and chief cells in precancerous lesions of gastric cancer: global knowledge mapping and emerging trends based on bibliometric analysis from 2004 to 2024
Source: Front Oncol. 2025 May 16;15:1556009. doi: 10.3389/fonc.2025.1556009 (PMC12122518; doi:10.3389/fonc.2025.1556009)
Supplement: Supplementary file 4 [file Table4.docx]

Table 4 The top 10 Key words associated with “PLGC-gastric stem cell” and “PLGC-chief cell”.

| Key words | Count | Centrality |
| --- | --- | --- |
| stem cells | 76 | 0.01 |
| intestinal metaplasia | 72 | 0.18 |
| cancer | 65 | 0.37 |
| gastric cancer | 59 | 0.16 |
| expression | 57 | 0.13 |
| helicobacter pylori | 55 | 0.17 |
| chief cells | 41 | 0.01 |
| stomach | 28 | 0.08 |
| polypeptide expressing metaplasia | 28 | 0.11 |
| helicobacter pylori infection | 26 | 0.05 |
